# Environmental conditions influence the biochemical properties of the fruiting bodies of *Tuber magnatum* Pico

**DOI:** 10.1038/s41598-018-25520-7

**Published:** 2018-05-08

**Authors:** Federico Vita, Flavio Antonio Franchina, Cosimo Taiti, Vittoria Locato, Giorgio Pennazza, Marco Santonico, Giorgia Purcaro, Laura De Gara, Stefano Mancuso, Luigi Mondello, Amedeo Alpi

**Affiliations:** 1 Department of Agriculture, Food and Environment, University of Pisa, Via Mariscoglio 34, 56124 Pisa, Italy; 2 Chromaleont Srl, c/o Department of Chemical, Biological, Pharmaceutical and Environmental Sciences Polo Annunziata, University of Messina, viale Annunziata, 98168 Messina, Italy; 3 LINV-Department of Plant Soil and Environmental Science, University of Florence, Viale delle idee 30, I-50019 Sesto-Fiorentino (FI), Florence, Italy; 4 Department of Medicine, Unit of Food Science and Nutrition, University “Campus Bio-Medico di Roma”, via Álvaro del Portillo 21, Rome, 00128 Italy; 5 Department of Engineering, Unit of Electronics for Sensor Systems, University “Campus Bio-Medico di Roma”, via Álvaro del Portillo 21, Rome, 00128 Italy; 6 Department of Chemical, Biological, Pharmaceutical and Environmental Sciences, Polo Annunziata, University of Messina, viale Annunziata, Messina, 98168 Italy; 7 A.R.E.A. Foundation, Via Tavoleria, 28, Pisa, 56125 Italy

## Abstract

The influences of various factors, including the symbiosis established with the roots of specific tree species, on the production of volatiles in the fruiting bodies of *Tuber magnatum* have not been investigated yet. Volatiles in *T. magnatum* fruiting bodies were quantitatively and qualitatively determined by both PTR-MS and GC-MS in order to compare the accuracy of the two methods. An electronic nose was also used to characterize truffle samples. The influence of environmental changes on the antioxidant capabilities of fruiting bodies was also determined. Statistically significant differences were found between fruiting bodies with different origins. The relationship between the quality of white truffle fruiting bodies and their specific host plant is described along with an analysis of metabolites other than VOCs that have ecological roles. Our results indicate that the geographical origin (Italy and Istria) of the fruiting bodies is correlated with the quantity and quality of volatiles and various antioxidant metabolites. This is the first report characterizing antioxidant compounds other than VOCs in white truffles. The correlation between geographical origin and antioxidant contents suggests that these compounds may be useful for certifying the geographical origin of truffles.

## Introduction

Gioacchini, *et al*.^1^ stated that a truffle’s aroma is a key contributor to the appreciation of its fruiting bodies. The aromatic compounds of fruiting bodies from several *Tuber* species have been analysed, and more than 200 volatile organic compounds (VOCs) have been studied^2^. The truffle aroma is derived from a complex mixture of substances from several chemical classes^3^. The VOCs emitted by truffles may play several roles similar to those reported in plants^4,5^ and may be heavily dependent on the environment. This means that VOCs may vary between individual truffles of the same species grown in different locations. Proton Transfer Reaction-Mass Spectrometry (PTR-MS) and multiple factorial analysis of the PTR signals have been used to analyse VOCs and to differentiate between samples of *Tuber magnatum* Pico fruiting bodies that were collected from two different regions of Italy^6^. Although the influence of truffles on the physiology of their host trees and on forest ecology has been described, little is known about the effect of the host plant on the VOCs produced by fruiting bodies.

Vita, *et al*.^6^ and Aprea, *et al*.^7^ both used PTR-TOF-MS to analyse white truffle aroma; the consistency of their results suggests that PTR-TOF-MS allows for the direct, accurate and rapid measurement of VOCs even at concentrations as low as a few parts per trillion by volume (pptv). Although PTR-MS is a powerful method, some compounds are ambiguously identified. To more completely describe the volatile compounds of white truffle, our analysis combined the highly sensitive PTR-MS method with gas-chromatography coupled to mass spectrometry (GC-MS) and extended the sampling of fruiting bodies to include various locations in Italy, taking into account the plant species associated with the fungus when possible.

The composition of the *T. magnatum* aroma is influenced by various factors including the presence of genetically differentiated populations^8^ and the variability of the bacterial^9^ and fungal^10,11^ communities associated with the truffle, but the climate of its geographical area of origin has the strongest effect^1^.

Antioxidants are another class of metabolites that vary a lot in response to the environment. They are chemically heterogeneous molecules that interfere with the levels of cellular reactive oxygen species that are generated within the cell as normal by-products of aerobic metabolism^12^. Antioxidant levels change in response to both biotic and abiotic environmental stresses because, similar to VOCs, they play a role in signalling and in responding to environmental changes. Phenols, ascorbate and glutathione are among the most studied antioxidants that have clear positive impacts on human health^13^ and delay the oxidative processes that occur in the decay and spoilage of food post-harvest. These metabolites are also involved in defending against biotic and abiotic environmental stresses^14^. There is considerable information on both the overall antioxidant capability and specific antioxidant molecules in plants and their relevance to plant growth and survival. However, there is currently little information for truffles, although some research is available on the correlation between phenols in truffles and antioxidant properties^15^. Variations in the contents of other antioxidant metabolites have also been reported in the desert truffle *Tirmania nivea* collected from different Middle Eastern regions^16^.

We hypothesized that antioxidant capability and the ascorbate and glutathione contents in truffles may be indicators of geographic origin or plant species association. Therefore, these parameters were analysed to evaluate the relevance of ecological influences on truffle growth.

The purpose of this research is to precisely determine the volatile compounds present in the aroma of *T. magnatum* fruiting bodies that were collected from various Italian and Istrian locations and to highlight VOCs that are specific to the different locations. We also attempted to distinguish fruiting bodies taken from the different locations by analysing antioxidant molecules that are influenced by environmental conditions.

## Results

### Quantitative and qualitative analysis of VOCs

#### PTR-TOF

Analysis of volatiles from *T. magnatum* was performed with PTR-TOF on selected fruiting bodies (Supplementary Information, Fig. S1, Table 1) and led to the putative identification of 66 compounds (Supplementary Information, Table S1). Analyses were focused on identifying VOCs ranging from 30–130 *m/z* in order to exploit the instrument’s high sensitivity for identifying smaller compounds. The identified compounds were grouped into chemical classes and then classified on the basis of their *m/z* ratio (both theoretical and measured), chemical name, and molecular formula. Percentages of VOCs reported in the Supplementary Information (Table S1) were calculated relative to the CAsa sample, (Casentino white willow, which emitted the highest amount of VOCs). To analyse the relationship between VOCs and geographical origin, the nine detected chemical classes (alcohols, AC; aldehydes, AD; aromatic compounds, AR; esters, ES; hydrocarbons, HC; ketones, KE; sulfur-containing compounds, SU; terpenes, TE; and others, OT) were treated as nine groups of variables in the statistical analyses. The data were then converted to a logarithmic scale using a log_10_ + 1 transformation, as described later in the Methods section.

**Table 1:** Sampling sites, host plants, and analyses performed. All fruiting bodies reached stage 5 of maturation as described in the Materials and Methods section. Identifier: the code used in the statistical analyses; Antiox. activity: the hydrophilic, hydrophobic, and total antioxidant activity assays; Antiox. comp.: glutathione, phenol and ascorbate assays; E-nose: electronic nose analysis.

Identifier	Site	Province	Region	Host plant	Antiox. activity	Antiox. comp.	E-nose	HS-GC	PTR-TOF-MS
ALqp	Alba	Cuneo	Piedmont	Sessile Oak (*Quercus petraea*)	x	x	x	x	x
ALpa	Alba	Cuneo	Piedmont	Poplar (*Populus alba*)				x	x
MScb	Sant’Angelo in Vado	Pesaro-Urbino	Marches	Hornbeam (*Carpinus betulus*)				x	x
MSqb	Sant’Angelo in Vado	Pesaro-Urbino	Marches	Downy Oak (*Quercus pubescens*)				x	x
MSqp	Sant’Angelo in Vado	Pesaro-Urbino	Marches	Sessile Oak (*Quercus petraea*)	x	x	x		x
MMcb	Mercatello sul Metauro	Pesaro-Urbino	Marches	Hornbeam (*Carpinus betulus*)				x	x
MMsa	Mercatello sul Metauro	Pesaro-Urbino	Marches	white Willow (*Salix alba*)				x	x
MMqc	Mercatello sul Metauro	Pesaro-Urbino	Marches	Turkey Oak (*Quercus cerris*)	x	x	x	x	x
MMpa	Mercatello sul Metauro	Pesaro-Urbino	Marches	Poplar (*Populus alba*)					x
CApa	Casentino	Florence/Arezzo	Tuscany	Poplar (*Populus alba*)	x	x	x	x	x
CAsa	Casentino	Florence/Arezzo	Tuscany	white Willow (*Salix alba*)	x	x	x	x	x
CAqp	Casentino	Florence/Arezzo	Tuscany	Sessile Oak (*Quercus petraea*)	x	x	x	x	x
SMwd	San Miniato	Pisa	Tuscany	n. s. (Wood)	x	x		x	x
HRwd	Levade	Istria	Croatia	n. s. (Wood)				x	x
SGwd	San Gimignano	Siena	Tuscany	n. s. (Wood)	x	x	x	x	x
ISpa	Isernia	Isernia	Molise	Poplar (*Populus alba*)	x	x	x	x	x
AQwd	Aquila	Aquila	Abruzzo	n. s. (Wood)	x	x	x		x

**Figure 1:**
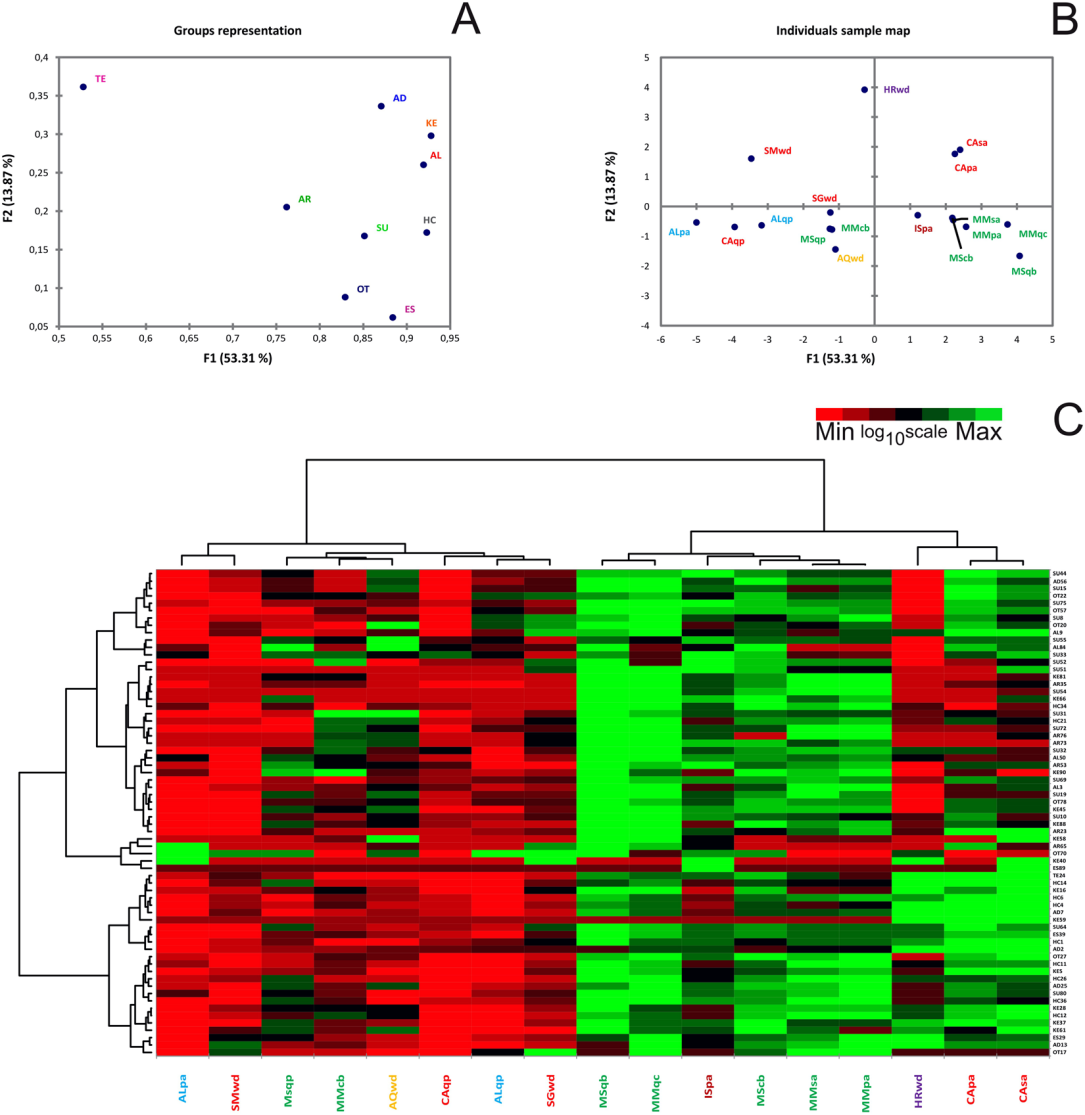
Multiple factor analysis (MFA) of VOCs analysed by PTR-TOF. F1 = first dimension, F2 = second dimension. Data were processed by log_10_ + 1 transformation. (**A**) Representation of groups of variables. Key codes: AL = Alcohols; AD = Aldehydes; AR = Aromatic compounds; ES = Esters; HC = Hydrocarbons; KE = Ketones; OT = Others; SU = Sulfur containing compounds; TE = Terpenes. (**B**) Representation of the selected samples in the multidimensional space of the MFA (F1, F2). The total inertia (*i.e*., total variance) included in the first two dimensions of the MFA was 67.19%. (**C**) Heat map based on the quantitative data obtained from PTR-TOF analysis. The samples analysed are listed in Table 1, while compounds identified through PTR-TOF analysis are reported in the Supplementary Information (Table S1). Data were log_10_ + 1 transformed.

MFA (Multiple Factor Analysis) revealed relationships among the PTR-TOF-MS fingerprints of the samples from the nine geographical areas. The coordinates of the nine groups of variables were used to create a map of the groups of compounds (Fig. 1A, Groups representation). These coordinates were calculated using the first two dimensions of the MFA (Dimensions 1 and 2 on the diagram), which included 67.18% of the total variance in the dataset. The contributions of the individual groups of variables to the first dimension of the MFA analysis (Table 2) generally ranged from 10.16% (aromatic compounds) to 12.38% (ketones), except for the terpene class, which only contributed 7.05% (although it included only one identified compound).

**Table 2:** Compound classes and their relative contribution to the MFA dimensions for the PTR-TOF analysis. Each dimension of a multivariate analysis can be described by the variables that are used to construct the factorial axes. ^l^Compound classes are sorted according to their relative contributions to dimension 1.

Compound class^l^	Dimension 1	Dimension 2
*Ketones*	12.38	15.28
*Hydrocarbons*	12.32	8.82
*Alcohols*	12.26	13.34
*Esters*	11.79	3.17
*Aldehydes*	11.62	17.24
*Sulfur containing compounds*	11.36	8.60
*Others*	11.06	4.51
*Aromatic compounds*	10.16	10.52
*Terpenes*	7.05	18.52

Different trends were evident in the contributions of each group of variables to the second MFA dimension. The contributions of terpenes (18.52%) and aldehydes (17.24%) were the most statistically significant. On the other hand, the contributions of esters (3.17%) and other compounds (4.51%) were low. The data from the MFA were also used to determine how samples were separated within the multidimensional space (Fig. 1B, Individuals factor map). Compounds that were significantly correlated (α = 0.05) to the two first dimensions of the MFA are reported in the Supplementary Information (Table S2). They were selected in relation to their statistical relevance for the first or the second dimension of MFA. Vinyl acetate (ES39) had the highest Pearson coefficient for axis 1 (0.922), while 4-methyl-3-pentene-2-one had the highest correlation coefficient for axis 2 (0.759).

The first (F1) and second (F2) factors explained 53.31% and 13.87% of the total variance, respectively. The spatial distributions of the samples in the multidimensional space of the MFA showed that the HRwd (Istria), SMwd (San Miniato), CAsa (Casentino white willow) and CApa (Casentino poplar) samples differed the most. Considering only the first dimension, the samples collected from the Piedmont region (ALqp, ALpa) were clearly distinct from most of the samples collected from the Marches and Molise regions. Furthermore, the association of mycorrhiza with oak was a common feature of samples from Alba, Casentino and Sant’Angelo in Vado (Fig. 1B).

Quantitative data were also visualized using heat maps (Fig. 1C), and two dendrograms were produced; one heatmap visualized the origins of the samples, and the other visualized VOC molecules. The data were clustered based on the quantity of compounds, which made it easy to compare patterns. The sample dendrogram (above the heatmap shown in Fig. 1C) grouped the truffles into three main clusters: the first included samples from the Piedmont, Tuscany, Abruzzo and Marche regions; the second included most of the samples from Marche and Molise; and the third cluster included samples from Tuscany and Croatia. The dendrogram on the left of the heatmap shows that the compounds clustered into three groups with no specific associations with their chemical classes.

#### GC-MS and GC-FID analyses

The GC-MS analyses of volatiles definitively identified 114 compounds, and 1 compound was tentatively identified (Supplementary Information, Table S3). The VOC profile was first characterized by GC-MS, and then, GC-FID was used for relative quantification. Absolute quantification of these compounds would have required the use of pure standards, but considering that no recognizable markers had been identified, we focused on improving our understanding of how the VOC profile related to the geographical origin by comparing VOC profiles from the different samples. Moreover, the SPME (Solid-Phase Microextraction) is not appropriate for quantitative absolute calibration.

**Figure 2:**
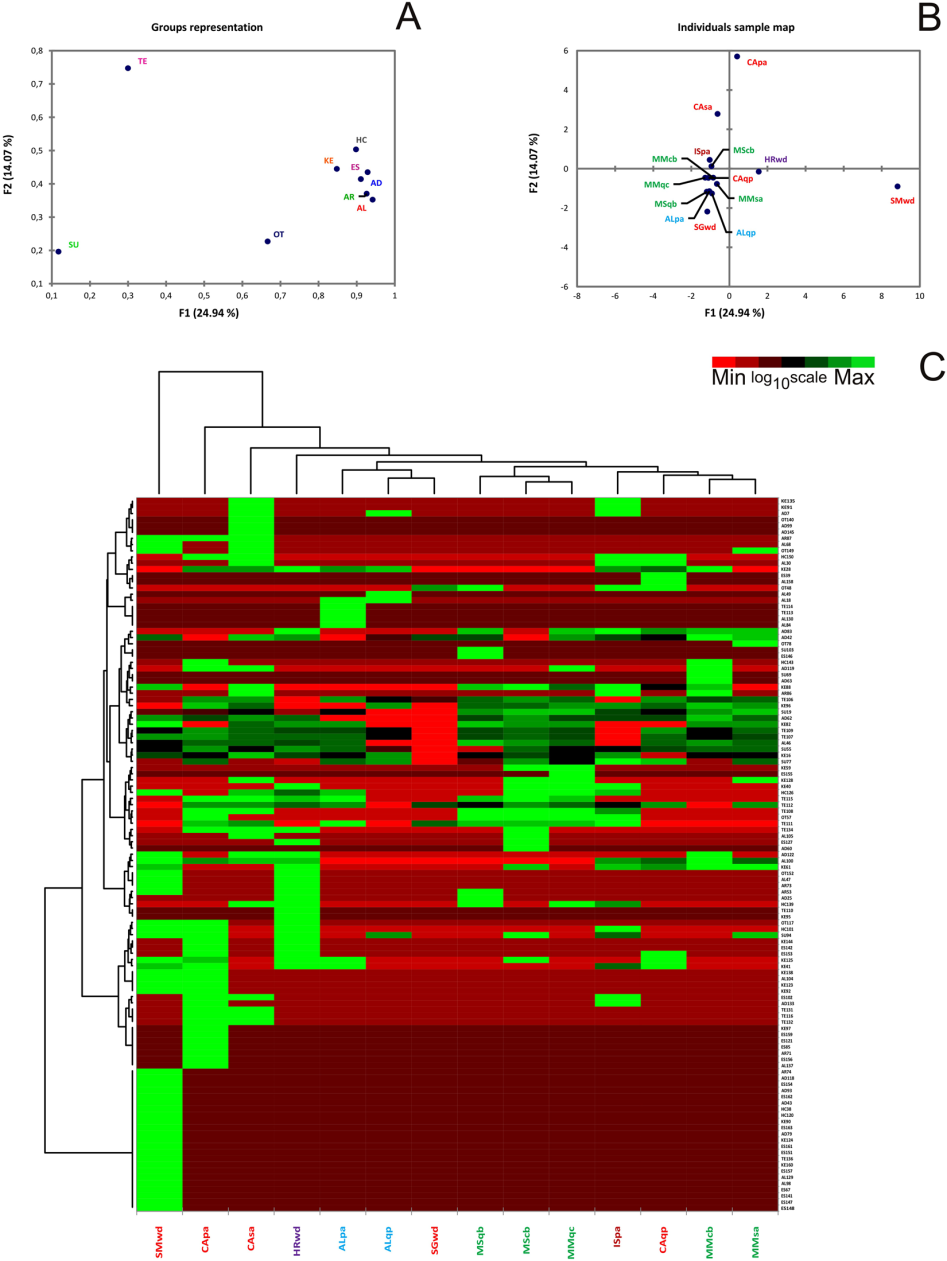
Multi factor analysis (MFA) of VOCs analysed by GC-MS. F1 = first dimension, F2 = second dimension. Data were log_10_ + 1 transformed. (**A**) Representation of groups of variables. Key codes: AL = Alcohols; AD = Aldehydes; AR = Aromatic compounds; ES = Esters; HC = Hydrocarbons; KE = Ketones; OT = Others; SU = Sulfur containing compounds; TE = Terpenes. (**B**) Representation of the selected samples in the multidimensional space of the MFA (F1, F2). The total inertia (*i.e*., total variance) included in the first two dimensions of the MFA was 39.01%. (**C**) Heat map based on the quantitative data obtained from GC-MS and GC-FID analysis. Samples are listed in Table 1, while compounds identified through GC-MS and GC-FID analysis are reported as Supplementary Information (Table S3). Data were log_10_ + 1 transformed.

The compounds identified and their percentages relative to the total VOCs found in the truffles are reported in the Supplementary Information (Table S3). Similar to what was done for PTR analyses, values are presented as percentages that were calculated relative to the sample with the highest VOC content (ISpa, Isernia white poplar), and the data for the nine chemical classes were analysed statistically (Supplementary Information, Table S3). The results of the MFA are shown in Fig. 2. Figure 2A shows that the contribution of the groups of compounds to the variation explained by the first dimension ranged from 1.80% for sulfur compounds to 14.41% for aldehydes (Table 3). Although terpenes and ‘other’ classes contributed relatively little to the variance, all of the other classes of compounds accounted for between 12.97% and 14.21% of the variance explained by the first dimension. The contributions to the variance in the second dimension were different. Terpenes accounted for the most variation (20.26%), and all of the other compound classes had relatively small contributions to the variation explained by the second dimension, with sulfur-containing compounds contributing the least (5.32%).

**Table 3:** Compound classes and their relative contribution to MFA dimensions for the GC-MS analysis. Each dimension of a multivariate analysis can be described by the variables that are used to construct the factorial axes. ^l^Compound classes are sorted according to their relative contributions to dimension 1.

Compound class^l^	Dimension 1	Dimension 2
*Aldehydes*	14.41	9.54
*Hydrocarbons*	14.21	11.78
*Alcohols*	14.16	10.03
*Esters*	13.93	11.22
*Aromatic compounds*	13.74	13.64
*Ketones*	12.97	12.06
*Others*	10.19	6.15
*Terpenes*	4.59	20.26
*Sulfur containing compounds*	1.80	5.32

The separation of samples in the multidimensional space of the MFA is shown in Fig. 2B. The CAsa and CApa samples were again easily distinguished, along with the sample from Isernia (ISpa). The first axis highlights the clear distinction between Alba and Marches samples and those from San Miniato.

Compounds that were significantly correlated (α = 0.05) to the two first dimensions are summarized in the Supplementary Information (Table S4), as was done for the PTR-TOF data. 3Z-octenol was positively correlated (0.958) with axis 1 along with four other compounds that had the same correlation coefficient. 3-octanone (−0.493) and β-pinene (−0.460) were negatively correlated with axis 1. The compound with the strongest positive correlation to axis 2 was α-terpinene (0.911), while 3-methylbutanal (−0.336) had the strongest negative correlation.

**Figure 3:**
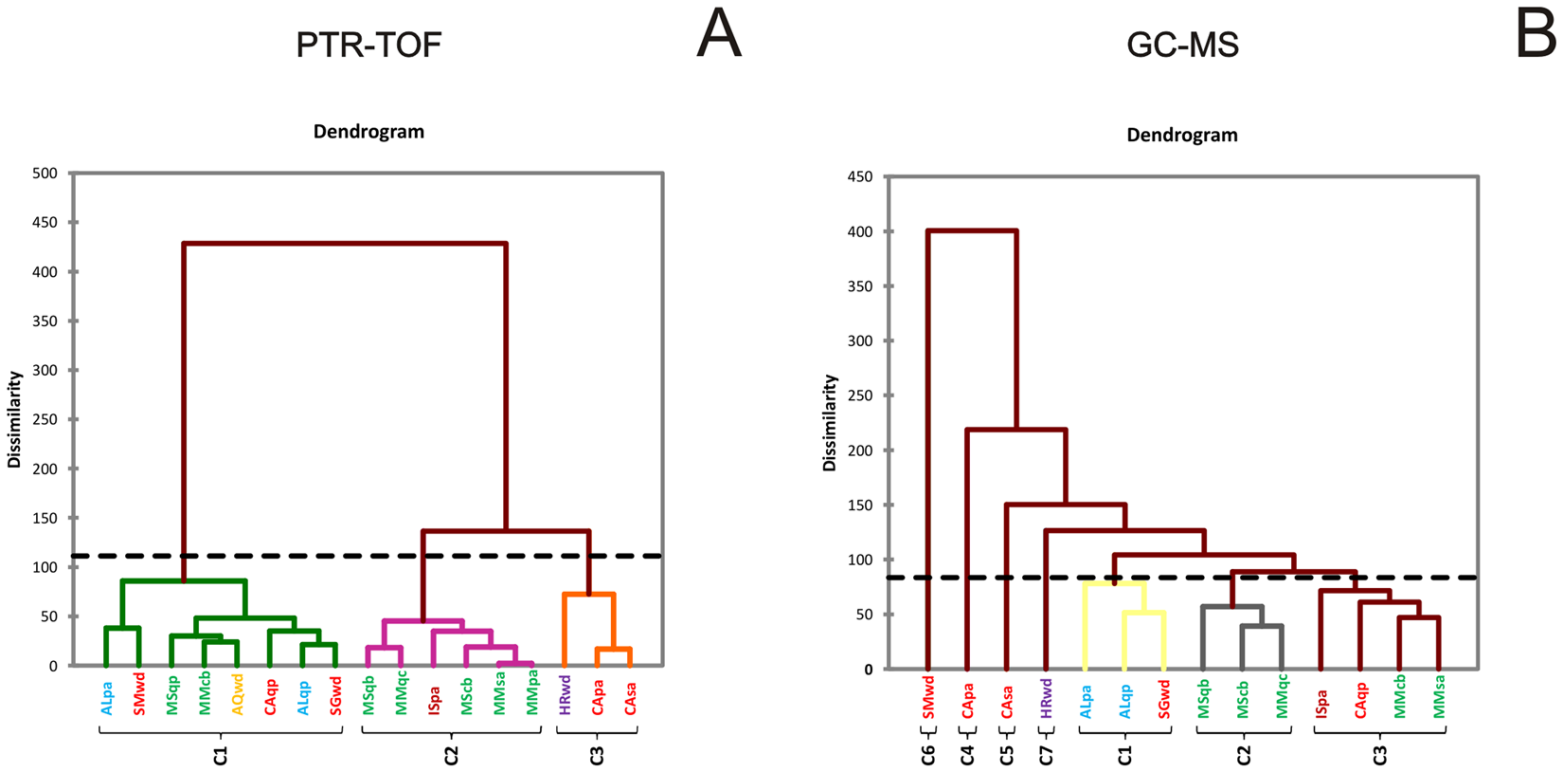
Results of aggregative hierarchical clustering (AHC) performed on PTR-TOF (**A**) and GC-MS data (**B**). C1-C7: Sample distribution classes, based on their dissimilarity coefficient. The dotted line represents the degree of truncation of the dendrogram used for creating classes and was automatically chosen based on the entropy level. The sample list is shown in Table 1.

The quantitative GC-MS data were also visualized with a heat map (Fig. 2C), as was done for the PTR-TOF data. The top dendrogram shows that the truffles clustered into seven main groups. The SMwd (San Miniato, wood) sample was the most distinct, followed by CApa (Casentino, poplar), CAsa (Casentino, white willow) and HRwd (Istria, wood). This distinction is more evident if the results of aggregative hierarchical clustering (AHC) are considered, which mirror the heat map results (Fig. 3). Unlike the PTR-TOF results in which samples were grouped into three clusters (Fig. 3A), clustering of the GC-MS results grouped samples into seven clusters, four of which contained only a single sample (Fig. 3B). Additional information for the AHC results is reported in the Supplementary Information (Table S5). The dendrogram on the left shows that compounds can be grouped into six clusters. One of these clusters mainly consists of esters and is made up of compounds only identified in the SMwd (San Miniato, wood) sample.

### Antioxidant properties

**Figure 4:**
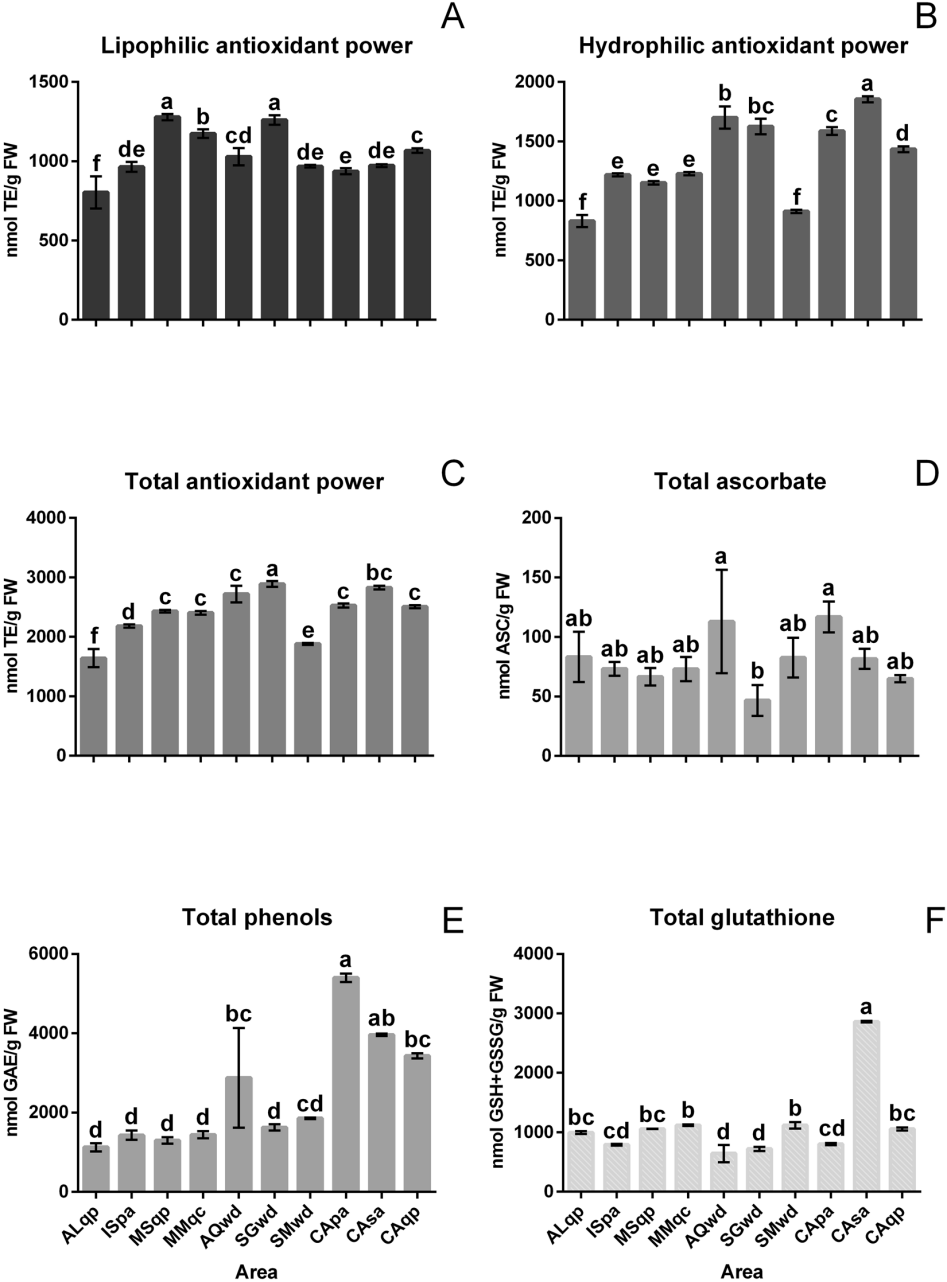
Antioxidant activity and relevant antioxidant compounds in the truffle samples. Tukey-HSD test was performed post hoc with a confidence level of 95% following an ANOVA. Values are the mean ± the standard deviation (n = 4). The sample details are shown in Table 1.

The hydrophilic, lipophilic and total antioxidant powers of the truffle samples were analysed. The fruiting bodies collected from different geographical areas had different ratios of lipophilic and hydrophilic antioxidant power. Fruiting bodies collected from Sant’Angelo in Vado (MSqp), Mercatello sul Metauro (MMqc) and San Gimignano (SGwd) had similar levels of lipophilic antioxidant power (Fig. 4A), while those coming from the other geographical areas had lower lipophilic antioxidant power. The hydrophilic antioxidant power (Fig. 4B) showed a different trend; the CAsa (Casentino, white willow) sample had the highest concentration of antioxidants, while the samples from Alba (ALqb) and San Miniato (SMwd) had the lowest values (less than 900 nmol TE/g of fresh weight). The total antioxidant power (Fig. 4C) was lowest in the ALqb truffle sample, while in all the other truffles, the antioxidant power was between 1880 and 2900 nmol TE/g of fresh weight. When the antioxidant powers of truffles collected in the same geographic area were compared in the same way as the samples collected from Casentino, their values were significantly different (Fig. 4C).

Analyses were also carried out for specific antioxidants including phenols, ascorbate and glutathione (Fig. 4D,E and F). The ascorbate content varied from approximately 46 nmoles/g of fresh weight in the fruiting bodies of SGwd to 116 nmoles/g of fresh weight in CApa fruiting bodies. Among the samples collected from the same areas, truffles from Marche (MMqc, MSqp) had ASC levels that were statistically similar, whereas the samples from Tuscany (SMwd, SGwd, CApa CAsa, CAqp) had more pronounced differences. The phenolic compound content varied from 1100 nmol of GAE/g of fresh weight to approximately 5400 nmol of GAE/g of fresh weight. The fruiting bodies collected in the Piedmont (ALqb), Marche (MSqp and MMqc) and Molise (ISpa) regions had similar levels of phenolic compounds but different to some samples collected from other regions. The GSH content varied among the samples from 644 nmol GSH/g of fresh weight in AQwd to 2863 nmol GSH/g of fresh weight in CAsa. The truffles that had sessile oak (CAqp, ALqp, MSqp) and poplar (CApa, ISpa) as their host plants had similar trends, but different values.

### RDA (Redundancy Analysis) results

**Figure 5:**
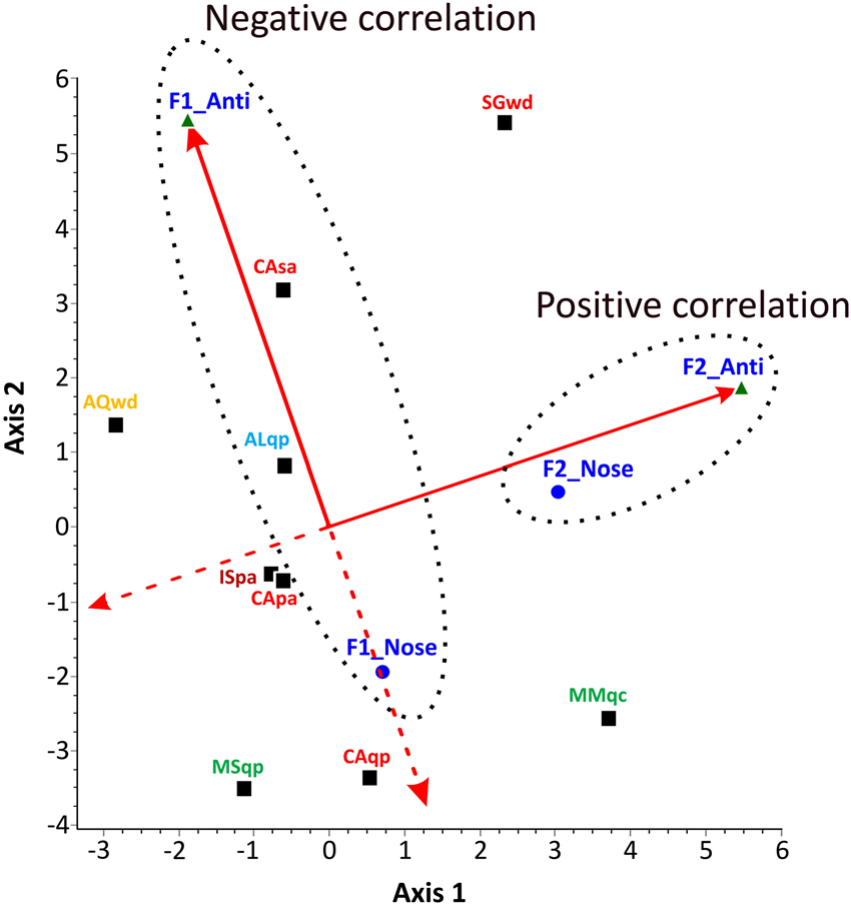
Triplot from RDA. The diagram displays three kinds of points: electronic nose data (circles markers), truffle samples (squares markers) and antioxidant parameters (triangles markers). The correlation of the antioxidant parameters with the RDA axes is represented by the length and directions of the red arrows. The truffle samples showing a high correlation with a given antioxidant parameter will be close to the position of the data. SMwd sample was not included because Nose data were not available. Nose = Electronic Nose; Anti = Antioxidant measurements.

Data obtained by electronic nose give a global information concerning all the volatiles compounds singularly detected by analytical methods. Similarly, the antioxidant capability represents the summation of the antioxidant effects of the single analysed metabolites. On this basis, RDA analysis was performed taking into account these parameters with the aim of detecting the existence of putative correlations between the parameters and between different parameters and truffle samples. Figure 5 shows the triplot from RDA. Deriving from the observation scores of the two original PCAs (Supplementary information, Fig. S1), the new observations (weighted average scores of the samples), electronic nose data (criterion variables) and antioxidant parameters (explanatory variables) are simultaneously represented in the triplot from the RDA.

**Figure 6:**
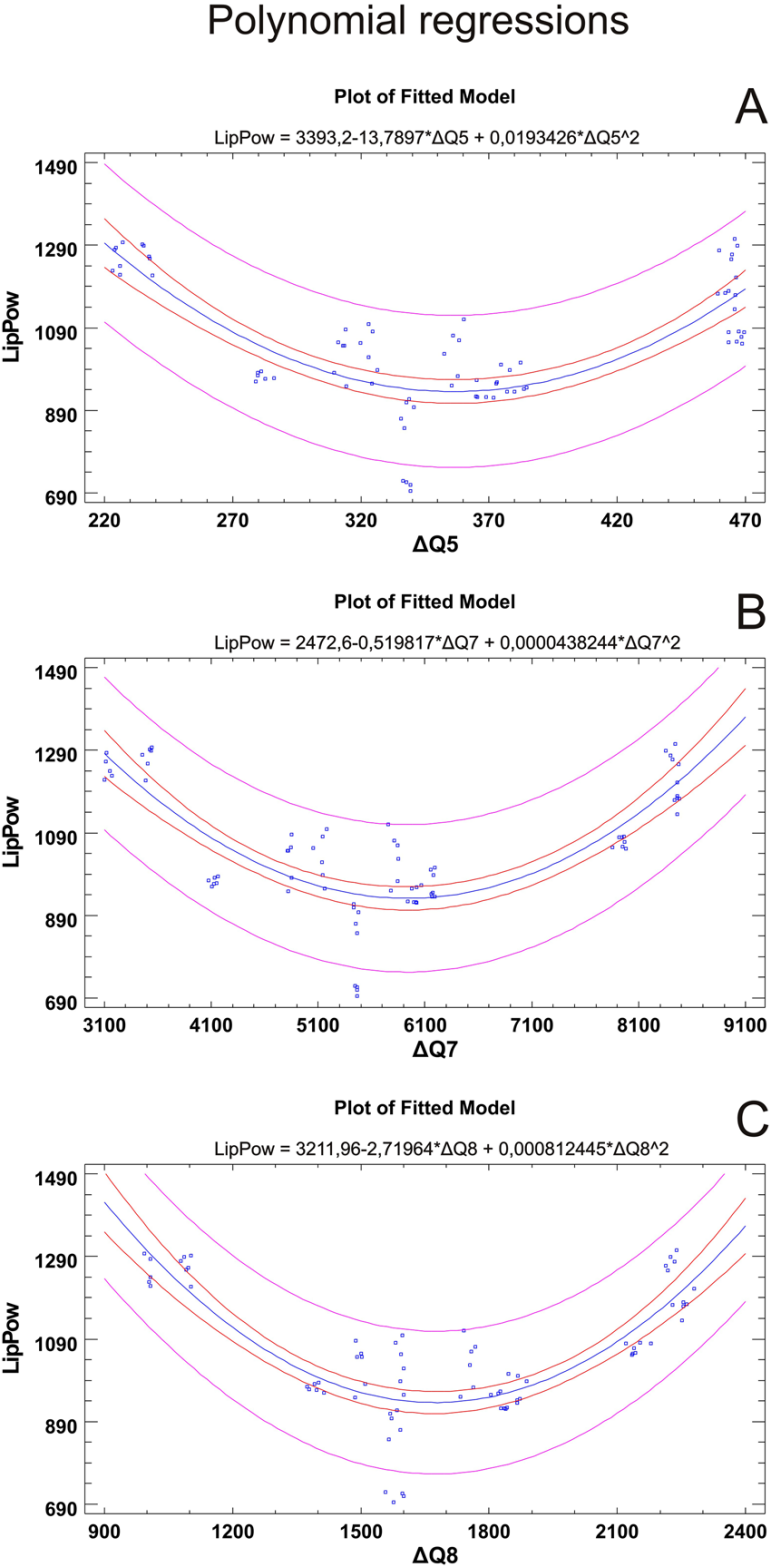
Polynomial regression plots (2^nd^ grade). Each plot shows the regression model for the statistically significant group of variables. The inner limits provide 95% confidence intervals for the mean value of y at any selected x. The outer lines are 95% prediction limits for new observations. Equations were reported for each fitted model. LipPow = Lipophilic antioxidant power.

In Fig. 5 we may identify the presence of specific correlations between the represented variables. F1_Anti and Fi_Nose were negatively correlated while the variables F2_Nose and F2_Anti reported a positive correlation. In addition, by observing the behavior of the accessions analyzed in relation to the variables, we can see different behaviors reported for samples collected from the same area (Casentino). Indeed, sample CAsa was positively correlated to the variable F1_Anti while sample CAqp and, to a lesser extent CApa, were positively correlated to the variable F1_Nose. When considering the same host plant, positive correlations were reported for sample ISpa and CApa (host plant; *Popolus alba*) and MSqp and CAqp (host plant; *Quercus petreae*); in this latter case, the only conflicting element is represented by the ALqp sample. A deeper statistical investigation of the relationships between single antioxidant parameters and electronic nose data was performed using polynomial regressions to explore correlations between each pair of variables, and calculated based on the whole dataset to highlight those that were most meaningful (Fig. 6). In particular, the results indicated that only data related to lipophilic antioxidant power (LipPow) fit with those related to Electronic nose data. Briefly, the second order polynomial models explain the 64.15% vs. ΔQ5, the 65.35% vs. ΔQ7 and the 68.33%. ΔQ8 of total variability.

## Discussion

In our experiment, samples differed in their geographical origin (which is the result of various climatic parameters including temperature and humidity) and in some cases in terms of the host plant from which they were collected. Rubini, *et al*.^8^ analysed *T. magnatum* from all over Italy and found that only the white truffle populations from the south (Benevento and Potenza) and those of northwestern Italy (Asti and Langhe) were genetically distinguishable. Samples from these regions were not included in our study. The climatic parameters for the various geographical areas are shown in the Supplementary Information (Table S6). It should be noted that during a five-year period (2012–2016), the average temperatures in the Casentino and Marche areas were the lowest, while San Miniato and San Gimignano had the highest temperatures; the temperature data for the two-year period in which samples were collected (2014–2015) are consistent with the five-year averages. Pluviometric data indicated that the most rainfall occurred in the Casentino and Marche areas, but there is considerable variability for the month of November when the truffles were harvested. However, the entire period prior - late spring, summer and early autumn - is more critical for fruiting body formation, and a severe drought during this time can lead to a loss of truffles.

The primary contribution of this work is a comprehensive profile of the VOCs generated by *T. magnatum* fruiting bodies. Such a profile had not previously existed for *T. magnatum*. In addition, some of the volatile compounds that were identified were associated with fruiting bodies from specific regions, and some compounds were unique to a specific origin. Environmental differences between the various sites include many factors, and one factor that possibly affects VOC content is the host-plant, although the impact of geographical origin is more significant.

### Sulfur-containing volatiles

The white truffle’s aroma is frequently described as sulfurous or garlicky, and this kind of odour is attributed to the presence of sulfur-containing VOCs. After the pioneering work of Fiecchi, *et al*.^17^, these VOCs were defined as “the most important group of odorants” and were detected in the fruiting bodies as well as in the mycelium of *T. magnatum* and other *Tuber* species^18,19^. These VOCs were also prevalent in our experimental analyses of *T. magnatum*, which is in agreement with the results from other studies on this truffle^7,20,21^.

Three of the compounds identified (dimethyl sulfide, dimethyl disulfide, and bis(methylthio)methane) were detected in all of the samples by GC-MS. Dimethyl trisulfide was found in the ALqp, MScb, CApa, SMwd, and HRwd samples. Therefore, these sulfides cannot be used to trace the origin of truffle fruiting bodies, but S-allyl-thiopropionate and 3-methylthio-propionaldehyde can. To our knowledge, these two volatile compounds have never been found in the aroma of any truffle species, but our analyses with PTR-TOF identified a higher total number of sulfur compounds.

### Aldehydes

Linoleic and linolenic acids are important components of biological membranes whose oxidative degradation by lipoxygenase produces aldehydes^22^. Many of these aldehydes are fundamental aroma components^23^. In our analysis, several of the aldehydes identified were specific to carpophores of definite origins. Aldehyde volatiles were mainly produced by Casentino samples, and they were also found to a lesser extent in Sant’Angelo in Vado fruiting bodies.

(4Z)-decenal was reported for the first time in orange essential oil^24^ but had never been identified as a *T. magnatum* volatile. In our experiments, it was emitted exclusively by the Casentino poplar (CApa) sample. The linear aldehyde *n*-octanal has rarely been reported in *T. magnatum*^25,26^ and was exclusive to the CAsa sample. The longer linear aldehyde *n*-undecanal (11 carbon atoms) is a common component of essential oils of *Citrus* spp. and fruit juice^27^, but it has never been found in truffles. We found that it was produced by the CAsa samples but not in any of the others.

(2E)-butenal has been detected in kiwi fruit essence and in other fruits^28^, but never in *Tuber* spp. Our analysis of the samples from MSqb and HRwd revealed that (2E)-butenal is produced in *T. magnatum* (although at a very low level), and this was confirmed by PTR-TOF results.

The only branched-chain aldehyde found in our analysis was 4-methylpent-2-enal in MScb. Consistent with the results of Belitz, *et al*.^22^, these aldehydes do not originate from lipid oxidation but from Strecker degradation of amino acids. The presence of the very similar compound (same molecular weight), 2-methylpent-2-enal, was detected in *Tuber borchii*^29^ and *T. magnatum*^25,26^. Other aldehydes, namely, 2-methyl pentanal, ligustral and benzaldehyde, occurred only in MMcb and SMwd samples. In a previous paper^6^, we reported that some volatile compounds are able to discriminate Alba from San Miniato samples. Some of these compounds (*e.g*., the aldehyde 2-methylbutanal) were also found in this analysis, but only in San Miniato fruiting bodies (Supplementary Information Table S3). This fact highlights the great potential of using VOCs to characterize truffle origin.

### Alcohols

Similar to the aldehydes, alcohols were specifically present in the aroma of fruiting bodies collected in Casentino.

In previous work, specific attention was given to the alcohol 1-octen-3-ol. For example, Combet, *et al*.^30^ stated that the eight carbon compounds are very important contributors to fungal aroma because they confer the characteristic mushroom odour. Splivallo, *et al*.^31^ also found them in several truffle species (*T. borchii* and *Tuber indicum*). 1-octen-3-ol is believed to act as signal to plants^2,19^. In our study, this compound was detected in fruiting bodies of various origins, and its occurrence was also not dependent on the host plant; hence, it is not useful for identifying a truffle’s origin.

Other alcohols were found to be linked to specific mycorrhiza (*e.g*., the C12 linear alcohol dodecanol in Casentino oak, CAqp). On the other hand, Casentino poplar (CApa) emitted both branched and linear volatiles as ethyl hexanol and (4Z)-decen-1-ol. Pennazza, *et al*.^26^ have reported the occasional occurrence of (4Z)-decen-1-ol in their stored white truffle fruiting bodies, which are naturally harvested in Marche, close to the Casentino area. In our analyses, this compound occurred only in CAsa and SMwd samples. SMwd, on the other hand, was the only sample to produce nonylol, a C-9 alcohol never found before in truffles. In our study, the volatile propyl alcohol was detected in the ALqp and ALpa samples, which has also never been reported in *T. magnatum*. Another alcohol, n-heptanol, was found only in ALpa by GC-MS but was revealed by PTR-TOF analysis to be in this sample as well as many others (although at a minimal level). The acquisition of data with two different instruments that have different specificities and sensitivities provided a greater degree of confidence in the final results.

### Ketones

The fatty acid β-oxidation pathway is also likely involved in the production of aromatic volatiles in mushrooms, including ketones and lactones^32^.

3-octanone occurs in various *Tuber* species and has been reported in *T. magnatum* fruiting bodies by several authors^7,20,26^; its presence was also confirmed by our results. Other ketones were produced only by fruiting bodies from a specific source and plant, such as CApa and CAsa. The ketone 2-octanone is a volatile that was found to be produced by *T. aestivum*^33^, and by *T. borchii* and *T. indicum*^31^.

*T. aestivum* and *T. uncinatum* are morphotypes of the same species caused by non-genetic environmental differences^34,35^. Splivallo, *et al*.^2^ studied C-8 compounds from fruiting bodies of *T. uncinatum*, and although they did not identify 2-octanone, it was clear that its production was influenced by mycorrhizal associations. For example, the C-8 VOCs were much more concentrated in hazel (*Corylus* spp.) mycorrhiza than in pine mycorrhiza.

In addition to the influence of the host plant, the truffle’s origin also determines which volatile compounds it produces. Splivallo, *et al*.^2^ reported that 3-octanone and 1-octen-3-ol are unique to the fruiting bodies of *T. uncinatum* of Italian origin and are not found in those from Switzerland and France.

We found the volatile compound 6-methyl-5-hepten-2-one in CApa and SMwd. This volatile compound has also been detected in *T. magnatum*^26^ but only at very low levels. We found a similar compound, 6-methyl-2-heptanone, was emitted by *T. melanosporum*. The ketone 2-decanone, a compound with an orange-like floral odor that has been detected in several plants^36^, has never been reported in *T. magnatum*. Another ketone, 2-undecanone, was almost specifically produced by CApa. This volatile has been detected in the aroma of several truffles, including *T. melanosporum* and *T. aestivum*^33^ and *T. indicum*^31^, but has never been found in *T. magnatum*. SMwd samples also had a specific ketone, 2-tridecanone, which has so far only been reported in field-grown onion^37^.

### Terpenes

Since PTR-TOF analysis detected only one terpene, our discussion is limited to the GC-MS data. For the PTR-TOF-MS data, compounds that have the same molecular formula cannot be reliably identified.

Volatile terpenes are major constituents of the plant volatilome and more importantly plant essential oils^4,38^. They have been investigated less in truffles, although their occurrences in the aromas of fruiting bodies have been reported^7,20,26,29^. However, they are often limited to very few compounds such as the cyclic monoterpene (molecular formula C_10_H_16_) limonene or the related alkyl benzene *p*-cymene.

In the most complete inventory of volatile compounds for fruiting bodies of three truffle species^31^, no molecules belonging to terpenes were reported. However, research on *T. magnatum* by Gioacchini, *et al*.^1^ indicated that white truffle aroma had a particular composition, which appeared to consist of only two main groups of volatiles: sulfur compounds and terpenes. In their study, which included fruiting bodies harvested in six Italian regions, they listed 28 sulfur compounds and 24 terpenes. Such discrepancies between reported data clearly show that a comprehensive description of the volatiles of white truffle is still far from complete.

Our results indicate that some terpenes are almost ubiquitous in the white truffle volatilome (*e.g*., limonene, and α- and β-pinene). However, other monoterpenes are uniquely produced by fruiting bodies of a specific origin or host-plant. Some monoterpenes, such as α-terpinene, eucalyptol and camphor, have been detected in Casentino fruiting bodies with mycorrhiza either on poplar (CApa) or on willow (CAsa). A wide variety of terpenes was found in the samples from Casentino, which agrees with the results of Gioacchini, *et al*.^1^. It is worth noting the proximity and the substantial similarity of the tree species that make up the forests of the Casentino and Marche areas where those authors collected their samples.

Sant’Angelo in Vado (MS) samples are from the Marche region. Additionally, the climatic data for the Casentino and Marche geographical areas are similar (Supplementary Information, Table S6). This suggests that the environment affects the aromatic composition and taste of white truffles and indicates the geographical origin - in this case, the Apennines in central Italy. The results of Vita, *et al*.^6^ indicate that the terpene class of compounds has the highest relative contribution to the second dimension of an MFA, thus highlighting the importance of these volatiles for describing the geographical origin of fruiting bodies.

### Hydrocarbons

Both PTR-TOF and GC-MS analyses detected hydrocarbons within the VOCs of *T. magnatum* fruiting bodies, although the compounds identified differed between the two methods.

The occurrence of this class of volatiles in the fruiting bodies of several truffle species, including white truffle, is well known^20,31,39^. In fact, Bellesia, *et al*.^25^ first reported the occurrence of decane from *T. magnatum* specimens collected in central Italy. Our results highlighted the almost ubiquitous occurrence of *n*-undecane, a substance detected in many fruits and vegetables^40^, as well as the unique occurrence of 1-decene in the fruiting bodies of samples from San Miniato (SMwd).

### Esters

As reported by Splivallo, *et al*.^31^, esters occur in the aroma of several species of truffles, but no esters have been reported in *T. magnatum* aroma. We found 14 esters primarily in the samples from Tuscany and Marche. Some of them, such as vinyl acetate, were uniquely produced by CAqp, which was confirmed by PTR-TOF.

Several esters were identified in the CApa fruiting bodies’ aroma: (2E)-hexenyl acetate is a common component of the volatilome of several fruits and mushrooms; ethyl lactate is a chemical that conveys a strawberry taste^41^; 3,5,5-trimethylhexyl-acetate is a volatile with a floral-sweet odour, and isobutyl pentanoate contributes to the odour of several apples^42^. Other volatile esters were detected specifically in the fruiting bodies from MMqc (*e.g*., allyl 3-methyl butoxyacetate) and from MSqb and MScb. 3-acetoxyoctane was identified for the first time in MSqb truffles. In MScb, isobutyl-isobutyrate, which is characterized by a pleasant fruity odour, was detected, and it is present in nearly all fruits.

The SMwd sample was particularly rich in esters; nine compounds were identified. This great variety of esters in San Miniato truffles is crucial for their characterization, and it may also play an important role in defining their aroma. Only low molecular weight esters, such as those found in the SMwd samples, have a pleasant odour. In particular, the low molecular-weight esters of butyric acid (similar to esters found in SMwd) have a pleasant aroma and taste^43^.

### Antioxidant profiling and the electronic nose

Antioxidant power is a measure of a compounds ability to detoxify reactive oxygen species produced within cells. Several molecules serve as antioxidants, of which phenols and polyphenols play a key role. It is worth noting that the methods used to extract metabolites from fruiting bodies do not result in the disruption of bacterial and yeast cells, which are the most abundant microorganisms colonizing fruiting bodies. Therefore, the profiles of antioxidant molecules that we have described are truly derived from truffle fruiting bodies. Unlike for plants, where many papers describe the antioxidant properties of different tissues and organs, there are very few studies on antioxidants in truffles or on the contribution of phenols to antioxidant activity in mushrooms^15,16,44^.

Our results suggest that phenols are not the main component conferring antioxidant properties to truffles, since no correlation was found between phenol content and antioxidant power in the fruiting bodies. The samples with the highest antioxidant powers were not the same as those with the highest phenol contents. This is in contrast with the results of Al-Laith^16^, who found a significant correlation between phenols and antioxidant capacity in the desert truffle *Tirmania nivea* collected from four different Middle Eastern regions^16^. In the truffles analysed here, neither the levels of glutathione nor those of ascorbate reflect the antioxidant power of the truffle fruiting bodies. This indicates that the total antioxidant activity is the result of various molecules present in different amounts that have different specificities for scavenging the various reactive species produced within cells.

A recent study reported that some polysaccharides strongly contribute to the antioxidant/redox activity of *Tuber huidongense*^45^. The amounts of ascorbate found in the fruiting bodies are much lower than those found in most of the metabolically active plant tissues, although there is considerable variability in antioxidant content between plant species, tissues and growth conditions^14^. Very small amounts of ascorbate have been found in desert truffles. In the fruiting bodies of *T. nivea*, the levels of ascorbate range between 5.9 and 11.4 mg/100 g of dry weight (which corresponds to 0.3 and 0.6 nmoles/g of dry weight)^16^. Similarly in *Terfezia boudieri*, the ascorbate content is 12.2 mg/100 g of dry weight (approximately 0.6 nmoles/g of dry weight)^46^. These values are more than 100–200 times lower than those found in *T. magnatum* (between 50 to 120 nmol/g of fresh weight). It should be emphasized that in these desert truffles, ascorbate was quantified using a colorimetric method based on the redox capability of ascorbate, while here, an enzymatic and indeed more specific method has been used. The presence of ascorbate metabolism in fungi is still under debate. Ascorbate has been reported in a few genera of yeast, but not in others^47,48^. According to a recent study on the evolution of the ascorbate biosynthetic pathways in eukaryotes, genes with homology to the sequences of the last enzymes in the eukaryotic (both photosynthetic and not-photosynthetic) ascorbate biosynthetic pathway are also present in some unicellular and multicellular fungi^49^. It is also known that heme-peroxidases (which catalyse reactions similar to plant ascorbate peroxidases) are widely distributed in all domains of life including fungi^50^. However, ascorbate was not detected in the fruiting bodies of the edible Basydiomicota *Pleurotus eryngii* when it was analysed with the same methods used here (De Gara and Locato, unpublished results). More in-depth studies are necessary in order to determine which phyletic lines of the Fungi kingdom are capable of ascorbate biosynthesis and how this ability was acquired by truffles. On the other hand, glutathione is a known part of truffle redox metabolism. Several glutathione-related genes have been identified in fungi including in *T. melanosporum* (the sequence of which is available at http://mycor.nancy.inra.fr/IMGC/TuberGenome/). It has also been suggested that glutathione appeared during evolution when oxygen increased in the atmosphere. It is likely that this was the first metabolic strategy for coping with oxygen-dependent oxidation^51^. Developmental phases could also strongly affect the levels of antioxidant metabolism as they do in other organisms, since both ascorbate and glutathione participate in the cell cycle and in other metabolic processes relevant to cell differentiation and organism growth^14^. This could be the focus of future studies.

Truffles collected in the same geographical area such as those from Alba and Aquila have some similarities in their lipophilic and hydrophilic antioxidant properties. However, the ecological environment where the truffles grow also has a strong influence on their antioxidant capability, since statistically significant differences were observed for antioxidant properties between samples that were collected in the same geographical area but had established mycorrhizal symbioses with different plants. This is even more evident in the phenol, ascorbate and glutathione contents, since their values varied significantly among the samples collected in the same area but near different plants. The results are in line with the physiological role of these metabolites in the organism / environment interaction. The climatic data are partially correlated with antioxidant contents. In fact, the highest level of hydrophilic antioxidant power and total glutathione was observed in the Casentino truffles associated with willow (CAsa), while total phenols were high in all Casentino truffles (Fig. 4).

The RDA results demonstrate how we could differentiate samples using two classes of independent variables. Similarly to Falasconi, *et al*.^52^, that have compared two different sets of data, we have used antioxidant measurements and electronic nose data together to differentiate samples.

The polynomial regression models show how the lipophilic antioxidant power is well correlated with the data of three nose sensors. It is worth noting that a consistent part of the identified VOC, such as terpens, are mostly lipophilic, therefore this correlation might suggest that part of the lipophilic antioxidant power is conferred by VOCs with lipophilic properties. Consistently, volatiles isoprenoids and phenylpropanes have been suggested to confer antioxidant protection under prolonged stress^53^.

Nevertheless, the use of a large number of samples could help to develop a system for identifying the origin of unknown truffles using this alternative, fast and reliable method which includes different kinds of analyses. In summary:

a) We used three methods (GC-MS, PTR-TOF, and electronic nose) to analyse the VOCs from white truffles, and to the best of our knowledge, we produced more accurate results than have previously been possible. Our findings also support the idea that volatiles, both individually and as a group of similar molecules, could be used to characterize the specific origin of truffle fruiting bodies. For example, a class of esters was unique to truffles from San Miniato, one of the Italian areas with higher quality white truffles, while acetyl isovaleryl and terpinolene were found in Istrian truffles (SMwd, HRwd - see Figs 1B and 2B).

b) The host plant has some influence on the types of volatile compounds emitted from truffles with the same origin. Alcohols and aldehydes such as decenol and decenal or n-octanal and n-undecanal are produced only by CApa and CAsa, respectively (see Figs 1B and 2B).

c) Our analysis of individual VOCs in *T. magnatum* indicates that sulfur compounds contribute the most to the formation of the truffle’s aroma (in particular, bis(methylthio)methane and dimethyl sulfide). Several VOCs belonging to the terpene class (*e.g*., limonene) are present specifically in the white truffles of central Italy.

d) The antioxidant properties of fruiting bodies that were collected from geographically distant areas were different, and this may be attributed to the specific mycorrhizal symbiosis (Fig. 4).

e) The RDA model appears useful to discriminate samples based on their geographical origin/ host plant. Further analysis confirmed the existence of a precise correlation between lipophilic antioxidant power and electronic nose results.

As a general comment, we consider it appropriate to mention that inside of the fruiting bodies, other fungi such as yeasts and bacteria can contribute to the biochemical features of truffles. Therefore, it is possible that some of the VOCs identified are not actually produced by the truffles.

## Methods

### Fruiting body sampling

Samples of *T. magnatum* Pico fruiting bodies were collected from various areas of Italy and Croatia (Supplementary Information, Fig. S2) during the second half of November in 2014 and 2015 at stage 5 of maturation^54^. Climatic data for the collection sites (5 years, 2012–2016) are included in the Supplementary Information (Table S6). Species identity was confirmed using two methods: microscopic observations and ITS analysis, as previously reported by Vita, *et al*.^6^. Five fruiting bodies were selected for analysis from a larger pool. For each sample group, five naturally growing fruiting bodies were harvested at 17 sites in specific regional areas, namely: three areas from Tuscany (San Gimignano, Casentino, San Miniato), two from Marches (Mercatello sul Metauro, Sant’Angelo in Vado), one from Piedmont (Alba), one from Molise (Isernia), one from Abruzzo (Aquila), and one from Croatia (Levade, Istria). The symbiotic plant species were known for several of the samples, including Casentino (*Populus alba*, *Salix alba*, *and Quercus petraea*); Mercatello sul Metauro (*Carpinus betulus*, *Salix alba*, *Quercus cerris*, and *Populus alba*); Sant’Angelo in Vado (*Carpinus betulus*, *Quercus pubescens*, and *Quercus petraea*); and Alba (*Quercus petraea* and *Populus alba*). Together, the total number of samples analysed was 85 (17 × 5) per year. The samples were analysed separately. Detailed sample information is shown in Table 1.

Three fruiting bodies were used for the MS analysis for each source and year (n = 6), and 2 fruiting bodies were used for the analysis of the antioxidant compounds for each origin and year (n = 4).

All samples were stored at 4 °C after collection, and all of the analyses were completed within three days. When it was not possible to comply with this requirement, even for a single year of experimentation, the samples were excluded from the analysis to avoid any alterations due to sample deterioration.

When possible, samples from the same geographical site were analysed on the same day.

### Mass spectrometry, antioxidant activity and antioxidant compound analysis

The full details for PTR-TOF, GC-MS/ GC-FID analysis, antioxidant activity, antioxidant compound analysis, PCA and electronic nose measurements are in the Supplementary Information Materials and Methods.

### Statistical analysis

Information related to the statistical analyses described in this article are reported in the Supplementary Information Materials and Methods.

## Electronic supplementary material

Supplementary Information
