# Supplementary material for: Environmental conditions influence the biochemical properties of the fruiting bodies of Tuber magnatum Pico
Source: Sci Rep. 2018 May 8;8:7243. doi: 10.1038/s41598-018-25520-7 (PMC5940868; doi:10.1038/s41598-018-25520-7)
Supplement: Supplementary file 1 — Supplementary Information [file 41598_2018_25520_MOESM1_ESM.docx]

Supplementary Information

**Environmental conditions influence the biochemical properties of the fruiting bodies of *Tuber magnatum* Pico.**

**Federico Vita^1^, Flavio Antonio Franchina^2^, Cosimo Taiti^3^, Vittoria Locato^4^, Giorgio Pennazza^5^, Marco Santonico^5^, Giorgia Purcaro^2^, Laura De Gara^4^, Stefano Mancuso^3^, Luigi Mondello^2,6^, Amedeo Alpi^1,7*^**

^1^Department of Agriculture, Food and Environment, University of Pisa, Via Mariscoglio 34, 56124, Pisa, Italy

^2^Chromaleont Srl, c/o Department of Chemical, Biological, Pharmaceutical and Environmental Sciences Polo Annunziata, University of Messina, viale Annunziata, 98168 Messina, Italy

^3^LINV-Department of Plant Soil and Environmental Science, University of Florence, Viale delle idee 30, I-50019 Sesto-Fiorentino (FI), Florence, Italy

^4^Department of Medicine, Unit of Food Science and Nutrition, University ‘‘Campus Bio-Medico di Roma’’, via Álvaro del Portillo 21, 00128 Rome, Italy

^5^Department of Engineering, Unit of Electronics for Sensor Systems, University “Campus Bio-Medico di Roma”, via Álvaro del Portillo 21, 00128 Rome, Italy

^6^Department of Chemical, Biological, Pharmaceutical and Environmental Sciences, Polo Annunziata, University of Messina, viale Annunziata, 98168 Messina, Italy

^7^A.R.E.A. Foundation, Via Tavoleria, 28 – 56125 Pisa, Italy

Author for correspondence: amedeo.alpi@gmail.com

**SI Materials and Methods**

**Volatile organic compounds analysis**

*PTR-TOF-MS analysis*

For each sample, five carpophores of about 10-15 g were stored at 4° C in glass vials and analyzed within 24 h. Sample preparation and VOCs monitoring were performed as described in [Vita, et al. ^6^](#_ENREF_6), while the raw data acquisition has been made following the procedure used by [Mancuso, et al. ^55^](#_ENREF_55)^,^[Taiti, et al. ^56^](#_ENREF_56). The day after, three of the five carpophores analyzed were used for GC-MS measurements and remaining two were further subjected to biochemical and electronic nose analysis. Analysis were mainly focused in the range from 30 to 120 *m/z*. Subsequently, we applied the empirical transmission curve to estimate the actual abundances of product ions over the product ions with mass range 30–150; the peak intensities for the different VOCs were converted in ppbv[^57^](#_ENREF_57)^,^[^58^](#_ENREF_58) using constant values for the reaction rate coefficient (k = 2.10^−9^ cm^3^ s^−1^) to calculate concentration for each VOC detected[^59^](#_ENREF_59)^,^[^60^](#_ENREF_60).

*SPME method*

The SPME method was adopted from a previous work[^26^](#_ENREF_26). Briefly, 1 g of fresh sample was placed in a 20 mL crimped vial. SPME extraction was carried out in the headspace mode (HS) using an autosampler AOC-5000 (Shimadzu), equipped with a fused silica fiber coated with a 50/30 μm layer of divinylbenzene/carboxen/polydimethylsiloxane, 1 cm long (Sigma-Aldrich/Supelco). The fiber was conditioned according to manufacturer’s instructions. Samples were conditioned for 5 min at 50°C, under agitation (clockwise, rotation at 500 rpm), before exposing the fiber for 20 min at 50°C, under continuous agitation. Analytes were then desorbed for 1 min at 260°C in the GC injector in splitless mode (1 min). Each sample was analyzed in triplicate.

*GC–MS and GC-FID analysis*

The GC–MS and GC-FID runs were carried out on a two parallel GC-QP2010 and GC2010 instruments (Shimadzu, Kyoto, Japan). The GC column used was a 30 m × 0.25 mm i.d. × 0.25 μm *d_f_* Supelcowax-10 column (Sigma-Aldrich/Supelco). Helium was used as carrier gas, at a constant linear velocity of 30.0 cm/s corresponding to an inlet pressure of 94.2 kPa for GC-MS and 97.4 kPa for GC-FID. Temperature program was the same in both analysis-type: 40°C at 3° C/min to 250°C, at 10°C/min to 270°C, held 10 min.

GC-MS ion source temperature was set at 200°C; the interface temperature, 250°C. Scan range was set to *m/z* 40–360, with a scanning rate of 1666 amu/s. FFSNC 3.01 (Shimadzu) and NIST11 (Wiley) commercial libraries were used for identification, applying two filters, namely a spectrum similarity match over 85% and Linear Retention Index (LRI) (related to a C_4_-C_24_ FAMEs mixture) agreement in the ±15 range (Supplementary information, Table S3). Confirmation of the identification was carried out also by comparison with reference standards.

The FID temperature was set at 280°C (sampling rate 40 ms) and gas flows were 40 mL/min for hydrogen and 400 mL/min for air, respectively.

The data handling was supported by GCMSsolution ver.2.6 and GCsolution software (Shimadzu) for GC-MS and GC-FID analysis, respectively.

**Determination of hydrophilic and lipophilic antioxidant capability**

Antioxidant capability (hydrophilic and lipophilic) of all texted truffle samples was assayed as scavenging activity toward 2,2’-azinobis-3-ethylbenzothiazoline-6-sulfonic acid (ABTS^●+^) monocationic radical according to the method described by [Re, et al. ^61^](#_ENREF_61) with slight modifications. Briefly the scavenger activity of the extracts toward ABTS^●+^ was spectrophotometrically monitored as decrease of absorbance at 734 nm.

The stock radical ABTS^●+^ solution was prepared dissolving 5.08 mM ABTS and 1.76 mM potassium persulfate in a buffer Na-P 0.1M pH 7.4. The solution was left overnight in the dark at room temperature. Before analysis, the stock solution was diluted in the buffer Na-P 0.1M pH 7.4 in 1:50 ratio (v/v).

Lipophilic fraction was obtained by homogenizing the sample in liquid nitrogen with acetone 100% in 1:5 ratio (p/v). Homogenate was centrifuged at 4°C, 5,000 g for 10 minutes. The recovered supernatant was used to analyze the lipophilic antioxidant capability.

The pellet obtained was dried, re-suspended in a phosphate buffer 50 mM pH 7.5 in 1:2 ratio (p/v) and centrifuged in the same conditions reported above. The recovered supernatant was used to analyze the hydrophilic antioxidant capability.

Hydrophilic and lipophilic antioxidant capabilities were determined by measuring the difference of absorbance registered in a time interval of 1 minute after sample addition (hydrophilic and lipophilic supernatant). A calibration curve of a standard antioxidant (Trolox) was obtained and hydrophilic and lipophilic antioxidant capabilities of samples were expressed as nmol of Trolox Equivalent (TE) for g fresh weight ± standard deviation of four different biological replicates.

*Determination of total phenol content*

Total phenol content of all tested truffle samples was determined according to [Waterhouse ^62^](#_ENREF_62). Total phenol content of samples was expressed as nmol of Gallic Acid Equivalent (GAE) for g of sample fresh weight of four biological replicates.

*Determination of ascorbate content*

Ascorbate (ASC) content of all tested truffle samples was determined by an enzymatic assay according to the method described by [Foyer, et al. ^63^](#_ENREF_63). This method, being based on the enzymatic ascorbate oxidation, was selected for its high specificity. ASC content was expressed as nmol of ASC for g fresh weight ± standard deviation of four different biological replicates. An extinction coefficient of 13.5 mM^-1^ ∙ cm^-1^ was considered.

*Determination of glutathione content*

Truffle samples were homogenized as described for ASC determination. Glutathione (GSH) pool (reduced plus oxidized forms) were assayed according to [De Pinto, et al. ^64^](#_ENREF_64) by using an enzymatic assay associated by spectrophotometric measurements. The total GSH amount was expressed as nmol of total GSH for g fresh weight ± standard deviation of four different biological replicates. A calibration curve in the range 0-30 μM ml^-1^ GSH was performed.

**Electronic nose**

BIONOTE, the gas sensor array (commonly dubbed electronic nose) used in this study was designed and fabricated by the Laboratory of Electronics for Sensor Systems of the Campus Bio-Medico University of Rome[^65^](#_ENREF_65). The sensors, quartz crystals oscillating at a resonance frequency of 20 Mhz in the thickness mode, were fabricated by CNR IMM Rome, Italy[^66^](#_ENREF_66). These transducers are functionalized with anthocyanins extracted from different plant tissues. Each material gives to each sensor unique adsorption properties in terms of sensitivity and specificity for a lot of volatile compounds. This permits to obtain a pattern of sensors because each sensor senses more compounds and each compound is sensed by more sensors.

BIONOTE is arranged in a chamber with a dedicated pneumatic system to efficiently deliver gas inside the chamber. In order to measure truffle samples headspace a dedicated sampling protocol has been designed[^26^](#_ENREF_26). The measurements outputs consist of differential values of frequency obtained considering the sensors interactions between a reference gas and samples mixture.

Because each sensor responds in different way the system provides a pattern of four sensors response. Each pattern represents a typical fingerprint of the sample.

**Statistical analysis**

*Mass spectrometry data*

Compounds identified in mass spectrometry analysis were classified on the base of their chemical and molecular properties, by using a unique code that includes the chemical class and a progressive number for PTR-TOF (Supplementary information, Table S1) as well as GC-MS data (Supplementary information, Table S3). The complete list of the identified compounds was reported as Supplementary information (Supplementary information, Table S7). To identify relationships among the selected samples based on data obtained both from PTR-TOF-MS than GC–MS and GC-FID analysis, multiple factorial analysis (MFA) was used[^67^](#_ENREF_67). The methodology of the MFA could be divided into two phases, as previously described by [Vita, et al. ^6^](#_ENREF_6). A log_10_ +1 transformation of the mass spectrometry data was performed with the aim to obtain a normal distribution of the values. Identified compounds were grouped into 9 classes depending on their chemical properties, AL, Alcohols; AD, Aldehydes; AR, Aromatic compounds; ES, Esters; HC, Hydrocarbons; KE, Ketones; OT, Others; SU, Sulfur containing compounds; TE, Terpenes. Volatile compounds significantly contributing to MFA dimensions were selected and then used to explain differences among truffles (compounds correlation coefficients, α = 0.05). Partial axes map related to the contribution of each class of compounds were also made through a vector representation that allows to see how the first two components generated by the first phase of the MFA analysis (the PCA calculated for each class of compounds) are related to Multiple Factor Analysis factors. The length and the direction of the vectors are directly correlated to their significance within each compound class.

Heat maps method was also used for visualizing mass spectrometry data sets organizing them as matrices. A heat map does two things to a matrix: first, it reorders the rows and columns so that rows (and columns) with similar profiles are closer to one another, rendering them to be more visible to the eye; second, each entry in the data matrix is displayed as a color, making it possible to view the patterns graphically[^6^](#_ENREF_6). The dendrograms were created using correlation-based distances and the Ward method of agglomeration was used in the present analysis[^68^](#_ENREF_68). Agglomerative hierarchical clustering (AHC) were performed to consolidate heat map results, using the same aforementioned parameters. Dendrograms truncation were performed according to default settings (entropy) to create homogeneous group of samples (classes) (Supplementary information, Table S5). All computations were performed using XLSTAT version 2014.5.03. Data reported for PTR-TOF (Supplementary information, Table S1) and GC–MS/ GC-FID (Supplementary information, Table S3) were normalized using a sample as internal standard (“reference sample”) to allow the comparison of different samples based on their composition. The referring sample was selected according to the higher sum of total signal intensities; CAsa (Casentino, white willow) and ISpa (Isernia, poplar) samples were respectively selected for PTR-TOF and GC–MS/ GC-FID data.

*Antioxidant capability data*

Statistical analysis was performed on all experimental data by means of GraphPad Prism statistical software program (6.01 version) using one-way analysis of variance (ANOVA) and non-parametric tests followed by Tukey’s HSD (honest significant difference) multiple comparison test. Differences with *p*-value of less of 0.05 (*p*<0.05) were considered to be statistically significant. Values marked by different letters were statistically different (*p*<0.05). Data were also analyzed using a PCA (principal component analysis) and their results were graphically processed to highlight the contribution and the relationships of each variable respectively (antioxidant compounds) in the truffle differentiation. PCA was performed using XLSTAT version 2014.5.03.

*Electronic nose data*

Electronic nose data were used for a PCA analysis. The analysis was computed on results related to 5 sensors. PCA was performed using XLSTAT version 2014.5.03.

*RDA analysis*

After performing PCAs for both the two groups of data (antioxidant parameters and electronic nose results), to have a first general overview of the possible correlations of the two sets of variables (Supplementary information, Fig. S1), a redundancy analysis (RDA) has been applied, involving the component scores obtained from both the two PCAs related to the two first principal components (F1_Nose and F2_Nose for the electronic nose results, F1_Anti and F2_Anti for the antioxidant parameters, respectively). RDA allows comparing two asymmetric groups of variables, extracting ordination axes that are linear combinations of antioxidant parameters variables explaining at the same time as much as possible of the variance in the electronic nose-based ordination of samples. The samples are ordered as in PCA, with the components maximally interpreting the electronic nose data as well[^69^](#_ENREF_69). Computations were performed by SYN-TAX 2000, Ordination package.

To deep the relationships between antioxidant parameters and electronic nose data, polynomial regressions for exploring correlations between each pair of variables were calculated at the 99% confidence level. Computations were performed using Statgraphics Centurion XVI.1.11.

**SI Results**

*PCA of antioxidant measurements*

PCA was performed on the antioxidant measurements (Supplementary information, Fig. S1), and the results showed that the first two dimensions (F1, F2) accounted for 76.25% of the total variance in the dataset. The contribution of each class of compounds to the variation is shown in Table S8A. The total antioxidant power and hydrophilic antioxidant power accounted for most of the variance (36.12% and 35.70%, respectively) for axis 1, while lipophilic antioxidant power and total ascorbate content accounted for 33.61% and 40.31% of the variance for axis 2.

The distribution of samples in the multidimensional space (Supplementary information, Fig. S1A) highlighted ALqp (Alba, sessile oak) and CAsa (Casentino, white willow) as the most notable samples on the horizontal PCA axis. Samples from Marche (MMqc, MSqp) and the samples from San Gimignano (SGwd) and Casentino (CAqp, Sessile oak) formed separate groups (lower left and lower right quadrants, respectively). The contribution of the individual classes of compounds is illustrated in the variable factor map in Figure S1B. It shows the relationships among the classes of antioxidant compounds that help differentiate the truffle samples. The antioxidant compounds (glutathione, phenols, and ascorbate) were positively correlated with antioxidant power (hydrophilic and total antioxidant power), while there was a negative correlation between ascorbate and lipophilic antioxidant power.

*PCA of electronic Nose data*

An electronic nose with four sensors was used to measure the fingerprints of volatile compounds from the nine truffle samples, and PCA was performed on the resulting data. Figure S1C shows that the first two principal components of the model accounted for 96.48% of the total variance in the dataset. Similar to the results from the antioxidant power analysis, the SGwd (San Gimignano, wood) sample was well distinguished in the multidimensional space of the PCA plot (Supplementary information, Fig. S1C). Samples from the same areas but with different host plants *(i.e.,* Casentino) were distinguishable on the first PCA axis, while samples from Marche (MSqp, MMqc) were distinguishable on the second axis. The contribution of each sensor value to the PCA is shown in the variable factor map in Supplementary information, Fig. S1D. Most of the values had a clear positive correlation except for ∆Q4, which strongly contributed to the second axis of the PCA (Supplementary information, Table S8B). On the other hand, the vector contributions for PCA axis 1 were generally equal.

**

**

**Fig. S1.** (*A*) Principal component analysis (PCA) of antioxidant power and antioxidant content. F1 = first dimension, F2 = second dimension; LIPO = lipophilic antioxidant power, HYDRO = hydrophilic antioxidant power, TOTAL L+H = total antioxidant power (hydrophilic and lipophilic), ASCO = total ascorbate, PHEN = total phenols, GLUT = total glutathione. (*B*) Correlation circle related to the contribution of each class of compounds. The length and the direction of the vectors are directly correlated to their significance. A positive correlation is indicated if the angle is less than 90 degrees, while a negative correlation is indicated if the angle reaches 180 degrees. There is no linear dependence if the angle is 90 degrees. The total inertia (*i.e.,* total variance) included in the first two dimensions of PCA was **76.25%**. (*C*) Principal component analysis (PCA) of electronic nose measurements. F1 = first dimension, F2 = second dimension. (*D*) Correlation circle related to the contribution of each class of variables. The length and the direction of the vectors are directly correlated to their significance. A positive correlation is indicated if the angle is less than 90 degrees, while a negative correlation is indicated if the angle reaches 180 degrees. There is no linear dependence if the angle is 90 degrees. Total inertia (*i.e.,* total variance) included in the first two dimensions of PCA was **96.48%**. Sample descriptions are shown in Table 1.

**
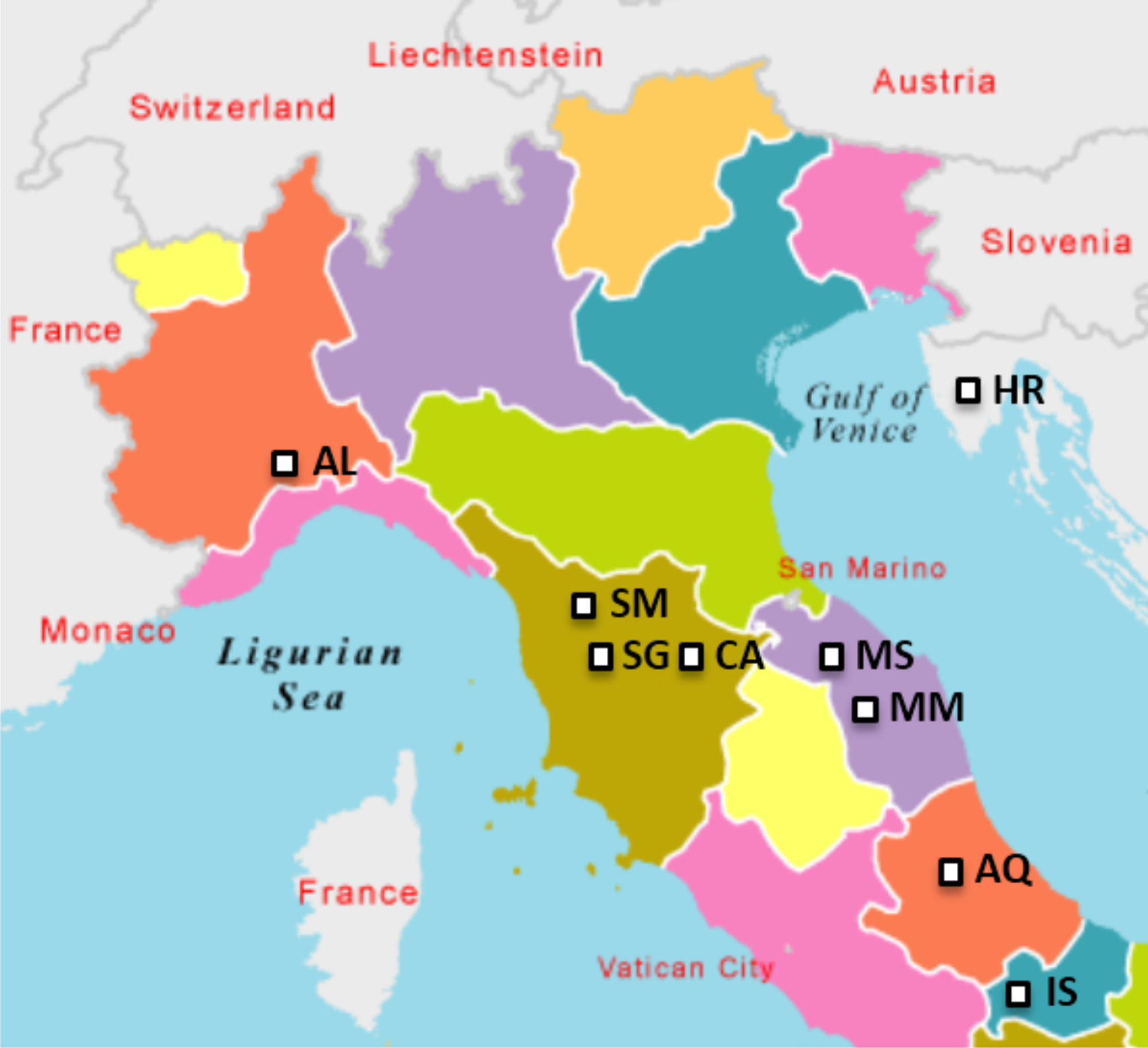
**

**Fig. S2.** Geographical localization of the selected samples listed in Table 1. This figure was modified and it is licensed under a Commons Attribution 3.0 Unported License (https://creativecommons.org/licenses/by/3.0/deed.en_US). The original version can be found here: [*http://www.mapsopensource.com/italy-states-outline-map.html*](http://www.mapsopensource.com/italy-states-outline-map.html)

**Table S1.** **Compounds identified through PTR-TOF-MS analysis: data are reported as medium normalized percentage (n=6)**. Sample CAsa (Casentino, white willow) was used as reference sample (sum of total signals=100) and other samples values were consequently calculated. Underlined compounds were identified in both GC-MS and PTR-TOF-MS.

Sample analyzed only with PTR-MS: MSqp (Sant’Angelo in Vado, sessile oak), MMpt (Mercatello sul Metauro. poplar) and AQwd (Aquila). ^a^ = Progressive code associated to MS analyses; ^b^ = Measured protonated *m/z* (H^+^); ^c^ =Theoretical protonated *m/z* (H^+^); # = compound quantity percentage < 0,01.

| **Unified code^a^** | **Putative Identifications** | **Chemical**  **Formula** | **Meas.**  ***m/z*^b^** | **Theor.**  ***m/z*^c^** | **ALqp** | **ALpa** | **MScb** | **MSqb** | **MSqp** | **MMcb** | **MMsa** | **MMqc** | **MMpa** | **CApa** | **CAsa** | **CAqp** | **SMwd** | **HRwd** | **SGwd** | **ISpa** | **AQwd** |
| --- | --- | --- | --- | --- | --- | --- | --- | --- | --- | --- | --- | --- | --- | --- | --- | --- | --- | --- | --- | --- | --- |
|  | **Sulfur-containing volatiles** |  |  |  |  |  |  |  |  |  |  |  |  |  |  |  |  |  |  |  |  |
| **8** | methanethial | **CH_2_S** | **46.990** | **46.995** | 18.74 | 1.09 | 6.85 | 18.74 | 3.06 | 4.49 | 15.04 | 23.82 | 22.50 | 17.01 | 20.82 | 6.91 | 2.76 | 4.66 | 32.13 | 10.47 | 10.57 |
| **10** | methanethiol | **CH_4_S** | **49.011** | **49.011** | 2.04 | 0.92 | 1.77 | 6.17 | 1.20 | 1.03 | 2.00 | 3.14 | 1.76 | 2.60 | 3.02 | 2.30 | 0.31 | 1.83 | 3.39 | 2.65 | 2.31 |
| **15** | Sulphur compounds (acetylenethiol / thiirene) | **C_2_H_2_S** | **58.993** | **58.995** | 0.01 | # | 0.02 | 0.05 | 0.01 | 0.01 | 0.02 | 0.03 | 0.02 | 0.04 | 0.06 | 0.02 | # | 0.03 | 0.04 | 0.03 | 0.01 |
| **19** | dimethyl sulfide | **C_2_H_6_S** | **63.026** | **63.026** | 19.79 | 7.63 | 14.59 | 33.08 | 9.37 | 5.98 | 11.40 | 24.44 | 16.59 | 30.31 | 33.49 | 12.13 | 6.60 | 2.99 | 26.20 | 20.04 | 24.93 |
| **31** | 1-propanethiol | **C_3_H_8_S** | **77.010** | **77.006** | 0.13 | 0.01 | 0.22 | 0.72 | 0.12 | 0.06 | 0.47 | 0.60 | 0.30 | 0.10 | 0.15 | 0.10 | 0.01 | 0.04 | 0.17 | 0.20 | 0.31 |
| **32** | Sulphur compounds (dithioformic acid) | **CH_2_S_2_** | **78.984** | **78.96** | 0.05 | # | 0.07 | 0.32 | 0.02 | 0.01 | 0.10 | 0.08 | 0.08 | 0.09 | 0.12 | 0.05 | 0.01 | 0.03 | 0.08 | 0.09 | 0.04 |
| **33** | dimethyl sulfoxide | **C_2_H_6_OS** | **79.021** | **79.021** | 0.01 | # | 0.02 | 0.08 | # | 0.09 | 0.02 | 0.04 | 0.02 | 0.02 | 0.03 | 0.01 | # | 0.01 | 0.03 | 0.03 | 0.10 |
| **44** | allyl methyl sulfide | **C_4_H_8_S** | **89.041** | **89.042** | - | - | # | # | # | # | # | # | # | # | # | # | - | # | # | # | # |
| **51** | 1,2-dithietane | **C_2_H_4_S_2_** | **92.980** | **92.983** | # | - | # | 0.03 | - | # | 0.01 | 0.01 | 0.01 | # | # | - | - | # | # | # | # |
| **52** | 2-methylmercaptoethanol | **C_3_H_8_OS** | **93.035** | **93.037** | # | # | # | # | # | # | # | # | # | # | # | # | - | - | # | # | # |
| **54** | dimethyl sulfone | **C_2_H_6_O_2_S** | **95.010** | **95.004** | 0.33 | 0.04 | 0.53 | 0.81 | 0.22 | 0.12 | 0.40 | 0.56 | 0.38 | 0.92 | 0.97 | 0.12 | 0.12 | 0.03 | 0.40 | 1.24 | 0.72 |
| **55** | dimethyl disulfide | **C_2_H_6_S_2_** | **95.199** | **94.998** | # | # | # | 0.01 | # | # | 0.01 | 0.01 | # | 0.01 | 0.01 | # | - | - | # | 0.01 | # |
| **64** | 2-methyl-4,5-dihydrothiophene | **C_5_H_8_S** | **101.040** | **101.042** | # | # | # | # | # | # | # | # | # | # | # | - | - | # | # | # | - |
| **69** | 3-methylthio-propionaldehyde | **C_4_H_8_OS** | **105.037** | **105.037** | - | - | # | 0.01 | - | - | # | # | # | # | # | - | - | - | # | # | - |
| **72** | 2-methylthioacetic acid | **C_3_H_6_O_2_S** | **106.995** | **107.016** | # | - | # | # | - | # | # | # | # | # | # | # | - | - | # | # | - |
| **75** | 2-ethynylthiophene | **C_6_H_4_S** | **109.010** | **109.011** | - | - | # | 0.02 | - | - | 0.01 | 0.01 | 0.01 | - | # | - | - | - | - | # | - |
| **80** | 2-methyl-3-furanthiol | **C_5_H_6_OS** | **115.020** | **115.021** | # | - | # | # | # | # | # | # | # | # | # | # | - | - | # | # | 0.01 |
|  | **Alcohols** |  |  |  |  |  |  |  |  |  |  |  |  |  |  |  |  |  |  |  |  |
| **3** | methanol | **CH_4_O** | **33.033** | **33.034** | 0.30 | 0.02 | 0.53 | 3.61 | 0.18 | 0.28 | 3.14 | 1.74 | 3.40 | 0.44 | 1.10 | 0.47 | 0.14 | 0.14 | 0.49 | 0.47 | 0.77 |
| **9** | ethanol | **C_2_H_6_O** | **47.049** | **47.049** | 0.05 | # | 0.05 | 0.13 | 0.01 | 0.02 | 0.04 | 0.14 | 0.06 | 0.18 | 1.00 | 0.01 | 0.03 | 0.07 | 0.27 | 0.05 | 0.06 |
| **50** | 2-3-butanediol | **C_4_H_10_O_2_** | **91.057** | **91.075** | - | # | # | 0.02 | # | # | # | 0.01 | # | # | # | # | - | # | # | # | # |
| **84** | *n*-heptanol | **C_7_H_16_O** | **117.078** | **117.073** | # | # | # | # | # | # | # | # | # | # | # | # | - | - | # | # | # |
|  | **Aldehydes** |  |  |  |  |  |  |  |  |  |  |  |  |  |  |  |  |  |  |  |  |
| **2** | formaldehyde | **CH_2_O** | **31.042** | **31.018** | 0.69 | 0.01 | 0.35 | 0.46 | 0.17 | 0.24 | 0.57 | 0.50 | 0.48 | 2.63 | 5.80 | 0.60 | 0.13 | 5.48 | 0.83 | 0.83 | 0.46 |
| **7** | acetic aldehyde | **C_2_H_4_O** | **45.033** | **45.033** | 0.04 | 0.03 | 0.20 | 0.58 | 0.02 | 0.06 | 0.22 | 0.24 | 0.30 | 0.64 | 1.03 | 0.12 | 0.08 | 7.07 | 0.11 | 0.13 | 0.11 |
| **13** | 2-propenal | **C_3_H_4_O** | **57.034** | **57.033** | # | # | 0.02 | 0.01 | # | 0.01 | 0.03 | 0.05 | 0.06 | 0.02 | 0.05 | # | 0.02 | 0.07 | 0.01 | 0.01 | # |
| **25** | (2E)-butenal | **C_4_H_6_O** | **71.049** | **71.049** | # | # | # | # | # | # | # | # | # | # | # | - | # | # | # | # | # |
| **56** | furfural (2-furancarboxaldehyde) | **C_5_H_4_O_2_** | **97.025** | **97.028** | 0.01 | # | 0.04 | 0.08 | 0.01 | # | 0.03 | 0.04 | 0.03 | 0.04 | 0.04 | 0.01 | # | # | 0.03 | 0.02 | 0.03 |
|  | **Aromatic compounds** |  |  |  |  |  |  |  |  |  |  |  |  |  |  |  |  |  |  |  |  |
| **23** | furan | **C_4_H_4_O** | **69.033** | **69.034** | # | # | # | 0.01 | # | # | # | # | # | 0.01 | 0.01 | # | - | # | # | # | # |
| **35** | 2-methylfuran | **C_5_H_6_O** | **83.049** | **83.049** | # | # | # | 0.02 | # | # | 0.02 | 0.01 | 0.03 | # | 0.01 | # | # | # | # | # | # |
| **53** | toluene | **C_7_H_8_** | **93.068** | **93.070** | - | # | # | # | # | # | # | # | # | # | # | # | - | - | - | # | # |
| **65** | ethynylbenzene | **C_8_H_6_** | **103.050** | **103.054** | - | # | - | # | - | - | - | # | - | # | # | - | - | - | # | # | # |
| **73** | ethylbenzene | **C_8_H_10_** | **107.086** | **107.086** | - | - | # | 0.01 | - | # | # | # | # | - | - | - | - | - | # | # | # |
| **76** | anisole | **C_7_H_8_O** | **109.065** | **109.065** | - | - | - | 0.01 | - | # | # | 0.01 | 0.01 | - | # | - | - | - | # | # | # |
|  | **Esters** |  |  |  |  |  |  |  |  |  |  |  |  |  |  |  |  |  |  |  |  |
| **29** | methyl acetate | **C_3_H_6_O_2_** | **75.044** | **75.044** | # | - | # | # | # | # | # | 0.02 | 0.01 | # | 0.01 | # | # | 0.01 | # | # | # |
| **39** | vinyl acetate | **C_4_H_6_O_2_** | **87.043** | **87.044** | # | - | # | 0.01 | # | # | # | # | # | # | 0.01 | # | - | # | # | # | # |
| **89** | methyl 2-Furancarboxylate | **C_6_H_6_O_3_** | **127.035** | **127.039** | - | - | - | - | - | - | - | - | - | - | # | - | - | - | - | # | - |
|  | **Hydrocarbons** |  |  |  |  |  |  |  |  |  |  |  |  |  |  |  |  |  |  |  |  |
| **1** | acetylene | **C_2_H_2_** | **27.025** | **27.023** | 1.94 | 0.14 | 1.69 | 8.43 | 0.92 | 0.33 | 3.58 | 2.58 | 3.15 | 7.11 | 17.83 | 2.10 | 0.46 | 5.46 | 5.30 | 3.69 | 0.76 |
| **4** | alkyl fragment | **C_3_H_4_** | **41.039** | **41.039** | 0.01 | 0.02 | 0.10 | 0.20 | 0.01 | 0.05 | 0.07 | 0.11 | 0.09 | 1.48 | 2.35 | 0.03 | 0.03 | 0.64 | 0.08 | 0.04 | 0.04 |
| **6** | alkyl fragment (propene) | **C_3_H_6_** | **43.054** | **43.054** | # | # | 0.05 | 0.14 | # | 0.02 | 0.08 | 0.06 | 0.12 | 0.33 | 0.82 | 0.02 | 0.01 | 0.11 | 0.02 | 0.02 | 0.02 |
| **11** | C4 compound | **C_4_H_4_** | **53.038** | **53.039** | # | # | 0.01 | 0.03 | # | # | 0.02 | 0.01 | 0.03 | 0.01 | 0.02 | # | # | 0.01 | # | # | # |
| **12** | alkyl fragment | **C_4_H_6_** | **55.054** | **55.054** | # | # | 0.12 | 0.14 | 0.02 | 0.05 | 0.17 | 0.07 | 0.23 | 0.12 | 0.37 | 0.01 | 0.01 | 0.08 | 0.04 | 0.04 | 0.06 |
| **14** | alkyl fragment (1-Butene) | **C_4_H_8_** | **57.068** | **57.070** | # | # | # | 0.01 | # | # | # | 0.01 | # | 0.02 | 0.05 | # | # | 0.02 | 0.01 | # | # |
| **21** | 3penten-1-yne | **C_5_H_6_** | **67.054** | **67.054** | 0.01 | # | 0.03 | 0.07 | - | 0.02 | 0.03 | 0.07 | 0.05 | 0.02 | 0.03 | # | # | 0.01 | 0.02 | 0.01 | 0.03 |
| **26** | alkyl fragment | **C_5_H_10_** | **71.086** | **71.086** | # | # | 0.01 | 0.01 | # | # | 0.01 | 0.01 | 0.02 | # | 0.01 | - | # | # | # | # | # |
| **34** | alkyl fragment (hexenals/hexenols/terpenoids) | **C_6_H_8_** | **81.069** | **81.070** | # | # | 0.01 | 0.05 | # | - | # | 0.01 | 0.01 | # | # | # | # | # | # | # | # |
| **36** | C6 compounds | **C_6_H_10_** | **83.085** | **83.086** | # | - | # | 0.01 | # | # | 0.01 | 0.01 | 0.01 | # | # | # | - | # | # | # | - |
|  | **Ketones** |  |  |  |  |  |  |  |  |  |  |  |  |  |  |  |  |  |  |  |  |
| **5** | alkyl fragment (ethenone) | **C_2_H_2_O** | **43.018** | **43.018** | 0.10 | 0.07 | 0.43 | 1.36 | 0.12 | 0.19 | 0.74 | 0.92 | 1.01 | 1.59 | 3.72 | 0.10 | 0.09 | 0.34 | 0.50 | 0.40 | 0.12 |
| **16** | acetone | **C_3_H_6_O** | **59.049** | **59.049** | 0.04 | 0.12 | 0.30 | 0.50 | 0.10 | 0.17 | 0.14 | 0.34 | 0.17 | 0.26 | 0.57 | 0.10 | 0.08 | 0.85 | 0.41 | 0.11 | 0.14 |
| **28** | butanone | **C_4_H_8_O** | **73.065** | **73.065** | # | # | 0.05 | 0.06 | 0.01 | 0.01 | 0.04 | 0.02 | 0.06 | 0.04 | 0.15 | # | # | 0.03 | 0.01 | 0.01 | 0.02 |
| **37** | 3-penten-2-one | **C_5_H_8_O** | **85.064** | **85.065** | # | - | # | 0.01 | # | # | # | # | # | # | # | # | - | # | # | # | # |
| **40** | 3-pentanone | **C_5_H_10_O** | **87.081** | **87.080** | - | - | # | 0.01 | # | # | # | # | # | # | 0.01 | - | - | # | # | # | # |
| **45** | 3-hydroxy-2-butanone | **C_4_H_8_O_2_** | **89.056** | **89.060** | - | # | - | - | - | - | - | - | - | - | # | - | - | # | # | # | - |
| **58** | 4-methyl-(5H)-furan-2-one | **C_5_H_6_O_2_** | **99.044** | **99.044j** | - | - | # | # | # | # | # | # | # | # | # | - | - | - | # | # | # |
| **59** | 4-methyl-3-pentene-2-one | **C_6_H_10_O** | **99.080** | **99.080** | # | # | # | # | # | # | # | # | # | # | # | # | - | - | # | # | # |
| **61** | acetylpropionyl | **C_5_H_8_O_2_** | **101.060** | **101.060** | - | - | - | # | - | # | # | # | # | - | # | - | - | - | - | # | # |
| **66** | 4-hydroxy-3-methyl-2-butanone | **C_5_H_10_O_2_** | **103.076** | **103.075** | - | - | - | - | - | - | - | - | - | # | # | - | - | # | - | - | - |
| **81** | 3,5-dimethyldiidro-2(3H)-furanone | **C_6_H_10_O_2_** | **115.075** | **115.075** | - | - | # | # | # | # | # | # | # | # | # | - | # | # | - | # | # |
| **88** | 2-acetyl-5-methylfuran | **C_7_H_8_O_2_** | **125.010** | **125.009** | - | - | # | # | # | - | # | # | # | - | # | - | - | - | - | # | - |
| **90** | 3-octen-2-one | **C_8_H_14_O** | **127.112** | **127.112** | - | - | # | 0.01 | # | # | 0.01 | 0.01 | 0.02 | - | # | - | - | - | - | # | - |
|  | **Terpenes** |  |  |  |  |  |  |  |  |  |  |  |  |  |  |  |  |  |  |  |  |
| **24** | 2-methyl-1,3- butadiene | **C_5_H_8_** | **69.070** | **69.070** | # | # | 0.01 | 0.01 | # | # | 0.01 | 0.01 | # | 0.03 | 0.06 | # | # | 0.03 | # | 0.01 | # |
|  | **Others** |  |  |  |  |  |  |  |  |  |  |  |  |  |  |  |  |  |  |  |  |
| **17** | acetic acid | **C_2_H_4_O_2_** | **61.028** | **61.028** | 4.07 | 1.08 | 2.56 | 2.17 | 0.71 | 0.93 | 5.66 | 10.37 | 6.37 | 1.82 | 3.19 | 0.85 | 2.14 | 2.21 | 12.50 | 1.98 | 0.96 |
| **20** | methanetriol | **CH_4_O_3_** | **65.023** | **65.023** | 1.96 | 0.23 | 0.81 | 2.07 | 0.28 | 0.26 | 0.94 | 2.29 | 1.34 | 2.30 | 3.01 | 0.47 | 0.52 | 0.45 | 3.28 | 0.93 | 2.95 |
| **22** | pyrrole | **C_4_H_5_N** | **68.050** | **68.049** | # | - | # | # | # | # | # | # | # | # | # | - | - | - | # | # | # |
| **27** | acrylic Acid | **C_3_H_4_O_2_** | **73.030** | **73.028** | # | # | 0.01 | 0.01 | # | # | 0.01 | 0.01 | 0.01 | 0.01 | 0.02 | # | # | # | 0.01 | 0.01 | 0.01 |
| **57** | 2,5-dimethyl-furan | **C_6_H_8_O** | **97.065** | **97.065** | # | - | # | # | - | # | # | 0.01 | # | # | # | - | - | - | # | # | # |
| **70** | 4-hydroxybutanoic acid | **C_4_H_8_O_3_** | **105.060** | **105.055** | 0.01 | 0.01 | # | # | # | - | - | # | - | # | # | - | # | # | 0.01 | # | # |
| **78** | 2-acetylfuran | **C_6_H_6_O_2_** | **111.044** | **111.043** | # | - | # | 0.02 | # | # | # | # | # | # | # | # | - | - | # | # | # |
|  |  |  |  |  | **ALqp** | **ALpa** | **MScb** | **MSqb** | **MSqp** | **MMcb** | **MMsa** | **MMqc** | **MMpa** | **CApa** | **CAsa** | **CAqp** | **SMwd** | **HRwd** | **SGwd** | **ISpa** | **AQwd** |
|  |  |  |  | **Total** | 50.36 | 11.47 | 31.48 | 80.34 | 16.61 | 14.46 | 45.10 | 72.46 | 58.82 | 70.22 | ***100.00*** | 26.56 | 13.58 | 32.71 | 86.42 | 43.61 | 45.59 |
|  |  |  |  |  |  |  |  |  |  |  |  |  |  |  |  |  |  |  |  |  |  |
|  |  |  |  |  |  |  |  |  |  |  |  |  |  |  |  |  |  |  |  |  |  |

**Table S2. Compounds significantly correlated (PTR-TOF analysis) to the first and second dimensions of the multiple factor analysis (MFA)**. Significant compounds were selected based on their correlation coefficients (α = 0.05) and sorted by Pearson correlation coefficient. Underlined compounds were also identified in GC-MS analysis.

| ***Tentative of Identifications*** | ***Chemical Formula*** | ***First dimension*** | ***Pearson correlation coefficient*** | ***Tentative of Identifications*** | ***Chemical Formula*** | ***Second dimension*** | ***Pearson correlation coefficient*** |
| --- | --- | --- | --- | --- | --- | --- | --- |
| *vinyl acetate* | **C_4_H_6_O_2_** | **ES39** | 0.922 | *4-methyl-3-pentene-2-one* | **C_6_H_10_O** | **KE59** | 0.759 |
| *acrylic Acid* | **C_3_H_4_O_2_** | **OT27** | 0.919 | *acetic aldehyde* | **C_2_H_4_O** | **AD7** | 0.652 |
| *alkyl fragment (ethenone)* | **C_2_H_2_O** | **KE5** | 0.914 | *alkyl fragment (1-Butene)* | **C_4_H_8_** | **HC14** | 0.612 |
| *C4 compound* | **C_4_H_4_** | **HC11** | 0.898 | *2-methyl-1,3- butadiene* | **C_5_H_8_** | **TE24** | 0.601 |
| *alkyl fragment* | **C_4_H_6_** | **HC12** | 0.895 | *ethylbenzene* | **C_8_H_10_** | **AR73** | -0.546 |
| *3-pentanone* | **C_5_H_10_O** | **KE40** | -0.108 | *dimethyl sulfoxide* | **C_2_H_6_OS** | **SU33** | -0.576 |
| *4-hydroxybutanoic acid* | **C_4_H_8_O_3_** | **OT70** | -0.252 | *3-octen-2-one* | **C_8_H_14_O** | **KE90** | -0.707 |

**Table S3. Compounds identified through GC-MS analysis: data are reported as medium normalized percentage (n=6)**. Sample ISpa (Isernia, Molise) was used as reference sample (sum of total signals=100) for quantification purposes and other samples values were consequently calculated. Underlined samples have been identified in both analyses (GC-MS/ PTR-TOF-MS). ^a^=Progressive code associated to MS analyses; ^b^= Compound tentatively identified only by GC-MS comparison; # = compound quantity percentage < 0.01.

| **Unified code^a^** | **Compounds** | **Experimental LRI** | **Reference**  **LRI** | **Chemical formula** | **Molecular weight** | **ALqp** | **ALpa** | **MScb** | **MSqb** | **MMcb** | **MMsa** | **MMqc** | **CApa** | **CAsa** | **CAqp** | **SMwd** | **HRwd** | **SGwd** | **ISpa** |
| --- | --- | --- | --- | --- | --- | --- | --- | --- | --- | --- | --- | --- | --- | --- | --- | --- | --- | --- | --- |
|  | **Sulfur-containing volatiles** |  |  |  |  |  |  |  |  |  |  |  |  |  |  |  |  |  |  |
| **19** | dimethyl sulfide | **304** | **296** | **C_2_H_6_S** | **62.134** | 0.07 | 7.42 | 25.17 | 16.37 | 17.17 | 25.68 | 20.54 | 5.24 | 6.46 | 6.23 | 3.55 | 3.00 | 0.80 | 9.15 |
| **55** | dimethyl disulfide | **469** | **465** | **C_2_H_6_S_2_** | **94.199** | 0.04 | 0.15 | 0.23 | 0.01 | 0.21 | 0.16 | 0.06 | 0.30 | 0.06 | 0.09 | 0.06 | 0.31 | - | 0.08 |
| **69** | 3-methylthio-propionaldehyde | **862** | **864** | **C_4_H_8_OS** | **104.170** | - | - | - | - | 0.08 | - | - | - | - | - | - | - | - | - |
| **77** | bis(methylthio)methane | **687** | **692** | **C_3_H_8_S_2_** | **108.226** | 57.78 | 46.39 | 54.06 | 26.33 | 20.91 | 51.36 | 37.57 | 48.35 | 22.63 | 71.44 | 23.55 | 19.98 | 3.36 | 85.94 |
| **94** | dimethyl trisulfide | **787** | **785** | **C_2_H_6_S_3_** | **126.260** | 0.02 | - | 0.08 | - | - | 0.03 | - | 0.80 | - | - | 0.07 | 0.08 | - | 0.01 |
| **103** | S-allyl-thiopropionate | **705** | **706** | **C_6_H_10_OS** | **130.210** | - | - | - | **#** | - | - | - | - | - | - | - | - | - | - |
|  | **Alcohols** |  |  |  |  |  |  |  |  |  |  |  |  |  |  |  |  |  |  |
| **18** | propyl alcohol | **441** | **434** | **C_3_H_8_O** | **60.100** | 0.16 | 0.33 | - | - | - | - | - | - | - | - | - | - | - | - |
| **30** | isobutyl alcohol | **494** | **480** | **C_4_H_10_O** | **74.122** | - | - | - | - | - | - | - | - | 0.15 | 0.18 | - | - | - | 0.02 |
| **46** | isopentyl alcohol | **619** | **618** | **C_5_H_12_O** | **88.148** | - | 0.06 | 0.11 | 0.21 | 0.10 | 0.08 | 0.08 | 0.05 | 0.07 | 0.08 | 0.01 | 0.03 | - | - |
| **47** | sec-pentanol | **514** | **514** | **C_5_H_12_O** | **88.148** | - | - | - | - | - | - | - | - | - | - | 0.11 | 0.02 | - | - |
| **49** | *sec*-butyl-carbinol | **617** | **611** | **C_5_H_12_O** | **88.148** | 0.03 | - | - | - | - | - | - | - | - | - | - | - | - | - |
| **68** | *n*-hexanol | **759** | **758** | **C_6_H_14_O** | **102.175** | - | - | - | - | - | - | - | - | 0.02 | - | 0.08 | - | - | - |
| **84** | *n*-heptanol | **863** | **854** | **C_7_H_16_O** | **116.201** | - | 0.02 | - | - | - | - | - | - | - | - | - | - | - | - |
| **98** | 3Z-octenol | **980** | **983** | **C_8_H_16_O** | **128.212** | - | - | - | - | - | - | - | - | - | - | 0.17 | - | - | - |
| **100** | 1-octen-3-ol | **860** | **858** | **C_8_H_16_O** | **128.220** | - | - | - | - | 0.11 | 0.01 | - | 0.03 | 0.05 | 0.01 | 0.26 | 0.04 | - | 0.02 |
| **104** | ethyl-hexanol | **897** | **900** | **C_8_H_18_O** | **130.230** | - | - | - | - | - | - | - | 0.06 | - | - | 0.04 | - | - | - |
| **105** | *n*-octanol | **956** | **959** | **C_8_H_18_O** | **130.230** | - | - | 0.30 | - | - | - | - | - | 0.53 | - | - | - | - | - |
| **129** | nonylol | **913** | **915** | **C_9_H_20_O** | **144.255** | - | - | - | - | - | - | - | - | - | - | 0.02 | - | - | - |
| **130** | 2-nonanol | **924** | **925** | **C_9_H_20_O** | **144.255** | - | 0.03 | - | - | - | - | - | - | - | - | - | - | - | - |
| **137** | (4Z)-decen-1-ol | **1191** | **1190** | **C_10_H_20_O** | **156.265** | - | - | - | - | - | - | - | 0.02 | - | - | - | - | - | - |
| **158** | dodecanol | **1360** | **1361** | **C_12_H_26_O** | **186.334** | - | - | - | - | - | - | - | - | - | 0.01 | - | - | - | - |
|  | **Aldehydes** |  |  |  |  |  |  |  |  |  |  |  |  |  |  |  |  |  |  |
| **7** | acetic aldehyde | **302** | **294** | **C_2_H_4_O** | **44.050** | 0.03 | - | - | - | - | - | - | - | 0.05 | - | - | - | - | 0.01 |
| **25** | (2E)-butenal | **449** | **440** | **C_4_H_6_O** | **70.090** | - | - | - | 0.02 | - | - | - | - | - | - | - | 0.20 | - | - |
| **42** | 3-methylbutanal | **362** | **368** | **C_5_H_10_O** | **86.130** | 0.02 | - | - | 0.09 | 3.07 | 0.94 | 0.43 | - | 0.65 | 0.03 | 0.14 | 0.37 | 0.05 | 0.06 |
| **43** | 2-methylbutanal | **360** | **366** | **C_5_H_10_O** | **86.132** | - | - | - | - | - | - | - | - | - | - | 0.04 | - | - | - |
| **60** | 4-methylpent-2-enal | **565** | **567** | **C_6_H_10_O** | **98.143** | - | - | 0.11 | - | - | - | - | - | - | - | - | - | - | - |
| **62** | *n*-hexanal | **479** | **477** | **C_6_H_12_O** | **100.159** | - | - | 0.30 | 0.05 | 0.60 | 0.16 | 0.06 | 0.08 | 0.34 | 0.06 | 0.27 | 0.13 | - | 0.08 |
| **63** | 2-methyl-pentanal | **421** | **417** | **C_6_H_12_O** | **100.159** | - | - | - | - | 0.03 | - | - | - | - | - | - | - | - | - |
| **79** | (2E)-heptenal | **733** | **728** | **C_7_H_12_O** | **112.170** | - | - | - | - | - | - | - | - | - | - | 0.10 | - | - | - |
| **83** | *n*-heptanal | **597** | **598** | **C_7_H_14_O** | **114.186** | - | - | - | 0.19 | 0.15 | 0.14 | 0.17 | - | - | 0.07 | - | 0.46 | - | 0.66 |
| **93** | (2E)-octenal | **840** | **842** | **C_8_H_14_O** | **126.196** | - | - | - | - | - | - | - | - | - | - | 0.08 | - | - | - |
| **99** | *n*-octanal | **697** | **698** | **C_8_H_16_O** | **128.212** | - | - | - | - | - | - | - | - | 0.02 | - | - | - | - | - |
| **118** | ligustral | **889** | **890** | **C_9_H_14_O** | **138.207** | - | - | - | - | - | - | - | - | - | - | 0.01 | - | - | - |
| **119** | trivertal | **840** | **843** | **C_9_H_14_O** | **138.207** | - | - | - | - | 0.23 | - | 0.03 | 0.03 | 0.24 | - | - | - | - | - |
| **122** | *n*-nonanal | **806** | **807** | **C_9_H_18_O** | **142.239** | - | - | - | - | 0.02 | - | - | - | 0.02 | - | 0.22 | 0.04 | - | - |
| **133** | (4Z)-decenal | **941** | **940** | **C_10_H_18_O** | **154.249** | - | - | - | - | - | - | - | 0.02 | - | - | - | - | - | **#** |
| **145** | *n*-undecanal | **1007** | **1007** | **C_11_H_22_O** | **170.292** | - | - | - | - | - | - | - | - | 0.05 | - | - | - | - | - |
|  | **Aromatic compounds** |  |  |  |  |  |  |  |  |  |  |  |  |  |  |  |  |  |  |
| **53** | toluene | **440** | **441** | **C_7_H_8_** | **92.138** | - | - | - | 0.14 | - | - | - | - | - | - | 0.65 | 0.40 | - | - |
| **71** | styrene | **658** | **666** | **C_8_H_8_** | **104.150** | - | - | - | - | - | - | - | 0.19 | - | - | - | - | - | - |
| **73** | ethyl-benzene | **525** | **525** | **C_8_H_10_** | **106.165** | - | - | - | - | - | - | - | - | - | - | 0.10 | 0.21 | - | - |
| **74** | benzaldehyde | **932** | **925** | **C_7_H_6_O** | **106.122** | - | - | - | - | - | - | - | - | - | - | 0.02 | - | - | - |
| **86** | phenylacetaldehyde | **1043** | **1044** | **C_8_H_8_O** | **120.149** | - | - | - | - | 0.21 | - | - | - | 0.02 | - | - | - | - | 0.03 |
| **87** | phenethyl alcohol | **1297** | **1306** | **C_8_H_10_O** | **122.160** | - | - | - | - | - | - | - | 0.08 | 1.01 | - | 0.04 | - | - | - |
|  | **Esters** |  |  |  |  |  |  |  |  |  |  |  |  |  |  |  |  |  |  |
| **39** | vinyl acetate | **400** | **393** | **C_4_H_6_O_2_** | **86.090** | - | - | - | - | - | - | - | - | - | 0.01 | - | - | - | - |
| **67** | methyl isobutyrate | **361** | **364** | **C_5_H_10_O_2_** | **102.132** | - | - | - | - | - | - | - | - | - | - | 0.12 | - | - | - |
| **85** | ethyl-lactate | **750** | **749** | **C_5_H_10_O_3_** | **118.130** | - | - | - | - | - | - | - | **#** | - | - | - | - | - | - |
| **102** | ethyl-acetoacetate | **865** | **863** | **C_6_H_10_O_3_** | **130.140** | - | - | - | - | - | - | - | 0.11 | 0.05 | - | - | - | - | 0.02 |
| **121** | (2E)-hexenyl acetate | **742** | **741** | **C_8_H_14_O_2_** | **142.196** | - | - | - | - | - | - | - | 0.06 | - | - | - | - | - | - |
| **127** | isobutyl-isobutyrate | **486** | **491** | **C_8_H_16_O_2_** | **144.211** | - | - | 0.14 | - | - | - | - | - | - | - | - | 0.02 | - | - |
| **141** | tetrahydrofurfuryl propionate | **1045** | **1044** | **C_8_H_14_O_3_** | **158.195** | - | - | - | - | - | - | - | - | - | - | 0.14 | - | - | - |
| **142** | isobutyl pentanoate | **860** | **862** | **C_9_H_18_O_2_** | **158.238** | - | - | - | - | - | - | - | 0.03 | - | - | - | 0.04 | - | - |
| **146** | 3-acetoxyoctane | **789** | **790** | **C_10_H_20_O_2_** | **172.265** | - | - | - | 0.11 | - | - | - | - | - | - | - | - | - | - |
| **147** | hexyl isobutyrate | **750** | **748** | **C_10_H_20_O_2_** | **172.265** | - | - | - | - | - | - | - | - | - | - | 0.13 | - | - | - |
| **148** | 3-methyl pentyl-Isobutyrate | **710** | **709** | **C_10_H_20_O_2_** | **172.265** | - | - | - | - | - | - | - | - | - | - | 1.40 | - | - | - |
| **151** | allyl caprylate | **977** | **979** | **C_11_H_20_O_2_** | **184.275** | - | - | - | - | - | - | - | - | - | - | **#** | - | - | - |
| **153** | (E)-2-nonen-1-yl acetate | **1043** | **1042** | **C_11_H_20_O_2_** | **184.275** | - | - | - | - | - | - | - | 0.10 | - | 0.10 | - | 0.03 | - | - |
| **154** | (6Z)-6-nonenyl acetate | **1031** | **1033** | **C_11_H_20_O_2_** | **184.275** | - | - | - | - | - | - | - | - | - | - | 0.27 | - | - | - |
| **155** | allyl 3-methyl butoxyacetate | **1047** | **1043** | **C_10_H_18_O_3_** | **186.251** | - | - | - | - | - | - | 0.83 | - | - | - | - | - | - | - |
| **156** | 3,5,5-trimethylhexyl-acetate | **816** | **814** | **C_11_H_22_O_2_** | **186.291** | - | - | - | - | - | - | - | 0.02 | - | - | - | - | - | - |
| **157** | heptyl-Isobutyrate | **855** | **857** | **C_11_H_22_O_2_** | **186.291** | - | - | - | - | - | - | - | - | - | - | 0.01 | - | - | - |
| **159** | allyl 3,5,5-trimethyl hexanoate**^b^** | **848** |  | **C_12_H_22_O_2_** | **198.302** | - | - | - | - | - | - | - | 0.03 | - | - | - | - | - | - |
| **161** | (E)-2-methyl-2-butenoic acid 1-ethylhexyl ester | **1056** | **1057** | **C_13_H_24_O_2_** | **212.330** | - | - | - | - | - | - | - | - | - | - | 0.12 | - | - | - |
| **162** | methyl-dodecanoate | **1199** | **1200** | **C_13_H_26_O_2_** | **214.344** | - | - | - | - | - | - | - | - | - | - | **#** | - | - | - |
| **163** | α-,α-dimethyl-phenethyl butyrate | **1301** | **1303** | **C_14_H_20_O_2_** | **220.307** | - | - | - | - | - | - | - | - | - | - | 0.03 | - | - | - |
|  | **Hydrocarbons** |  |  |  |  |  |  |  |  |  |  |  |  |  |  |  |  |  |  |
| **38** | cyclohexane | **309** | **301** | **C_6_H_12_** | **84.160** | - | - | - | - | - | - | - | - | - | - | 0.02 | - | - | - |
| **101** | *n*-nonane | **357** | **364** | **C_9_H_20_** | **128.255** | - | - | - | - | - | - | - | 0.05 | - | - | 0.03 | 0.03 | - | 0.02 |
| **120** | 1-decene | **446** | **444** | **C_10_H_20_** | **140.266** | - | - | - | - | - | - | - | - | - | - | 0.07 | - | - | - |
| **126** | *n*-decane | **412** | **406** | **C_10_H_22_** | **142.280** | - | 0.09 | 0.47 | - | - | - | 0.11 | - | 0.07 | - | 0.24 | 0.01 | - | 0.04 |
| **139** | *n*-undecane | **493** | **492** | **C_11_H_24_** | **156.308** | - | - | - | 0.22 | - | - | 0.18 | - | 0.11 | - | - | 1.22 | - | 0.01 |
| **143** | 1-dodecene | **651** | **657** | **C_12_H_24_** | **168.320** | - | - | - | - | 0.04 | - | - | 0.17 | - | - | - | - | - | - |
| **150** | 1-tridecene | **756** | **759** | **C_13_H_26_** | **182.346** | - | - | - | - | - | - | - | 0.17 | 0.05 | 0.23 | - | - | - | 0.04 |
|  | **Ketones** |  |  |  |  |  |  |  |  |  |  |  |  |  |  |  |  |  |  |
| **16** | acetone | **308** | **297** | **C_3_H_6_O** | **58.079** | 1.89 | 0.24 | 0.66 | 0.33 | 0.35 | 0.34 | 0.37 | 0.35 | 3.00 | 0.03 | 0.61 | 0.59 | - | 1.73 |
| **28** | butanone | **357** | **350** | **C_4_H_8_O** | **72.106** | 0.10 | 0.06 | - | - | 0.87 | - | - | 0.05 | 0.07 | 0.02 | - | 1.05 | - | 0.07 |
| **40** | 3-pentanone | **397** | **388** | **C_5_H_10_O** | **86.132** | - | - | 0.19 | - | - | - | 0.14 | - | - | - | - | 0.13 | - | 0.02 |
| **41** | 2-pentanone | **398** | **397** | **C_5_H_10_O** | **86.130** | - | 0.16 | - | - | - | - | - | 0.11 | - | 1.59 | 0.05 | 0.10 | - | **#** |
| **59** | 4-methyl-3-pentene-2-one | **526** | **530** | **C_6_H_10_O** | **98.143** | - | - | 0.10 | - | - | - | 0.50 | - | - | - | - | - | - | - |
| **61** | acetylpropionyl | **453** | **456** | **C_5_H_8_O_2_** | **100.120** | - | - | 0.02 | - | 0.04 | 0.03 | - | - | - | 0.01 | 0.02 | 0.06 | - | 0.03 |
| **82** | 2-heptanone | **592** | **597** | **C_7_H_14_O** | **114.186** | - | 0.04 | 0.05 | 0.11 | 0.04 | 0.04 | 0.03 | - | 0.02 | - | 0.20 | 0.03 | - | - |
| **88** | 2-acetyl-5-methylfuran | **1018** | **1020** | **C_7_H_8_O_2_** | **124.139** | - | - | 0.78 | 0.35 | 0.33 | - | 0.05 | - | 1.31 | 0.01 | 0.42 | - | - | 1.53 |
| **90** | 3-octen-2-one | **816** | **816** | **C_8_H_14_O** | **126.196** | - | - | - | - | - | - | - | - | - | - | 0.03 | - | - | - |
| **91** | 2-acetyl-cyclopentanone | **1009** | **1012** | **C_7_H_10_O_2_** | **126.153** | - | - | - | - | - | - | - | - | 0.13 | - | - | - | - | 0.01 |
| **92** | 6-methyl-hept-5-en-2-one | **745** | **746** | **C_8_H_14_O** | **126.196** | - | - | - | - | - | - | - | 0.03 | - | - | 0.04 | - | - | - |
| **95** | acetyl isovaleryl | **551** | **552** | **C_7_H_12_O_2_** | **128.169** | - | - | - | - | - | - | - | - | - | - | - | 0.15 | - | - |
| **96** | 3-octanone | **667** | **671** | **C_8_H_16_O** | **128.212** | 0.12 | - | 0.05 | 0.08 | 0.29 | 0.10 | 0.12 | 0.29 | 0.07 | 0.06 | - | - | - | 0.12 |
| **97** | 2-octanone | **695** | **696** | **C_8_H_16_O** | **128.212** | - | - | - | - | - | - | - | 0.08 | - | - | - | - | - | - |
| **123** | diisobutyl ketone | **584** | **587** | **C_9_H_18_O** | **142.239** | - | - | - | - | - | - | - | 0.01 | - | - | 0.01 | - | - | - |
| **124** | 4-nonanone | **734** | **734** | **C_9_H_18_O** | **142.239** | - | - | - | - | - | - | - | - | - | - | 0.95 | - | - | - |
| **125** | 2-nonanone | **797** | **802** | **C_9_H_18_O** | **142.239** | - | 0.16 | 0.09 | - | - | - | - | 0.09 | - | 0.38 | 0.54 | 0.20 | - | - |
| **128** | butyroin | **974** | **972** | **C_8_H_16_O_2_** | **144.211** | - | - | 0.54 | - | - | 0.66 | 1.46 | - | 1.14 | - | - | - | - | - |
| **135** | γ-nonalactone | **1418** | **1419** | **C_9_H_16_O_2_** | **156.222** | - | - | - | - | - | - | - | - | 0.08 | - | - | - | - | 0.02 |
| **138** | 2-decanone | **903** | **906** | **C_10_H_20_O** | **156.265** | - | - | - | - | - | - | - | 0.08 | - | - | 0.04 | - | - | - |
| **144** | 2-undecanone | **1003** | **1006** | **C_11_H_22_O** | **170.292** | - | - | - | - | - | - | - | 0.09 | - | - | - | 0.01 | - | - |
| **160** | 2-tridecanone | **1207** | **1213** | **C_13_H_26_O** | **198.345** | - | - | - | - | - | - | - | - | - | - | **#** | - | - | - |
|  | **Terpenes** |  |  |  |  |  |  |  |  |  |  |  |  |  |  |  |  |  |  |
| **106** | *para*-cymene | **677** | **678** | **C_10_H_14_** | **134.218** | 0.05 | 0.49 | 0.56 | 0.30 | 0.15 | 0.36 | 0.28 | 0.66 | 0.45 | 0.57 | 0.01 | - | 0.04 | - |
| **107** | γ-terpinene | **652** | **654** | **C_10_H_16_** | **136.234** | 0.12 | 0.79 | 0.64 | 0.30 | 0.12 | 0.32 | 0.26 | 1.00 | 0.67 | 0.86 | 1.19 | 0.46 | - | - |
| **108** | sabinene | **516** | **518** | **C_10_H_16_** | **136.234** | - | - | 0.08 | 0.06 | - | - | 0.06 | 0.12 | 0.09 | - | - | - | - | 0.01 |
| **109** | limonene | **610** | **608** | **C_10_H_16_** | **136.234** | 1.87 | 2.78 | 7.47 | 3.55 | 1.91 | 4.30 | 3.05 | 11.97 | 7.65 | 9.90 | 1.25 | 2.49 | - | 0.01 |
|  |  |  |  |  |  |  |  |  |  |  |  |  |  |  |  |  |  |  |  |
| **110** | terpinolene | **684** | **681** | **C_10_H_16_** | **136.234** | - | - | - | - | - | - | - | - | - | - | - | 0.03 | - | - |
| **111** | α-pinene | **424** | **427** | **C_10_H_16_** | **136.234** | - | 0.15 | 0.11 | 0.07 | - | - | 0.06 | 0.07 | 0.04 | - | - | - | 0.01 | 0.18 |
| **112** | β-pinene | **503** | **505** | **C_10_H_16_** | **136.234** | - | 0.27 | 0.39 | 0.04 | - | 0.25 | 0.17 | 0.50 | 0.40 | 0.36 | - | 0.12 | 0.05 | 0.03 |
| **113** | 2,2-dimethyl-5-methylene norbornane | **460** | **464** | **C_10_H_16_** | **136.234** | - | 0.42 | - | - | - | - | - | - | - | - | - | - | - | - |
| **114** | α-fenchene | **452** | **454** | **C_10_H_16_** | **136.238** | - | 0.11 | - | - | - | - | - | - | - | - | - | - | - | - |
| **115** | myrcene | **568** | **567** | **C_10_H_16_** | **136.240** | - | 0.30 | 0.15 | 0.07 | - | - | 0.07 | 0.25 | 0.16 | - | - | 0.07 | - | - |
| **116** | α-terpinene | **587** | **586** | **C_10_H_16_** | **136.240** | - | - | - | - | - | - | - | 0.04 | 0.02 | - | - | - | - | - |
| **131** | camphor | **920** | **918** | **C_10_H_16_O** | **152.233** | - | - | - | - | - | - | - | 0.03 | 0.05 | - | - | - | - | - |
| **132** | eucalyptol | **617** | **614** | **C_10_H_18_O** | **154.249** | - | - | - | - | - | - | - | 0.02 | 0.20 | - | - | - | - | - |
| **134** | linalool | **955** | **956** | **C_10_H_18_O** | **154.249** | - | - | 0.08 | - | - | - | - | 0.04 | 0.06 | - | - | 0.04 | - | - |
| **136** | α-dihydro-terpineol | **996** | **992** | **C_10_H_20_O** | **156.265** | - | - | - | - | - | - | - | - | - | - | 0.04 | - | - | - |
|  | **Others** |  |  |  |  |  |  |  |  |  |  |  |  |  |  |  |  |  |  |
| **48** | tert-butyl methyl ether | **290** | **284** | **C_5_H_12_O** | **88.148** | - | - | - | 0.05 | - | - | - | - | - | 0.17 | - | - | **#** | 0.03 |
| **57** | 2,5-dimethyl-furan | **382** | **384** | **C_6_H_8_O** | **96.127** | - | - | 0.08 | 0.01 | - | - | 0.13 | 0.01 | - | - | - | - | - | 0.01 |
| **78** | 2-acetylfuran | **913** | **917** | **C_6_H_6_O_2_** | **110.110** | - | - | - | - | - | 0.65 | - | - | - | - | - | - | - | - |
| **117** | 2-pentyl-furan | **632** | **638** | **C_9_H_14_O** | **138.207** | - | - | - | - | - | - | - | 0.05 | - | - | 0.03 | 0.14 | - | - |
| **140** | *n*-butyric anhydride | **1202** | **1200** | **C_8_H_14_O_3_** | **158.195** | - | - | - | - | - | - | - | - | 0.01 | - | - | - | - | - |
| **149** | diethyl acetal | **361** | **360** | **C_10_H_24_O_2_** | **176.296** | - | - | - | - | - | 0.10 | - | - | 0.68 | - | 0.04 | - | - | - |
| **152** | (Z)-3-nonenyl acetate | **1008** | **1010** | **C_11_H_20_O_2_** | **184.275** | - | - | - | - | - | - | - | - | - | - | 0.13 | 0.04 | - | - |
|  |  |  |  |  |  | **ALqp** | **ALpa** | **MScb** | **MSqb** | **MMcb** | **MMsa** | **MMqc** | **CApa** | **CAsa** | **CAqp** | **SMwd** | **HRwd** | **SGwd** | **ISpa** |
|  |  |  |  |  | **Total** | 62.3 | 60.5 | 93.0 | 49.0 | 47.0 | 85.7 | 66.8 | 71.9 | 48.9 | 92.5 | 37.8 | 32.3 | 4.3 | ***100.0*** |

**Table S4. Compounds significantly correlated (GC-MS analysis) to first and second dimensions of the multiple factor analysis (MFA).** Significant compounds were selected based on their correlation coefficients (α = 0.05) and sorted by Pearson correlation coefficient.

| ***Compounds*** | ***Chemical Formula*** | ***First dimension*** | ***Pearson correlation coefficient*** | ***Compounds*** | ***Chemical Formula*** | ***Second dimension*** | ***Pearson correlation coefficient*** |
| --- | --- | --- | --- | --- | --- | --- | --- |
| *3Z-octenol* | **C_8_H_16_O** | **AL98** | 0.958 | *α-terpinene* | **C_10_H_16_** | **TE116** | 0.911 |
| *nonylol* | **C_9_H_20_O** | **AL129** | 0.958 | *camphor* | **C_10_H_16_O** | **TE131** | 0.894 |
| *tetrahydrofurfuryl propionate* | **C_8_H_14_O_3_** | **ES141** | 0.958 | *eucalyptol* | **C_10_H_18_O** | **TE132** | 0.853 |
| *methyl-dodecanoate* | **C_13_H_26_O_2_** | **ES162** | 0.958 | *ethyl-acetoacetate* | **C_6_H_10_O_3_** | **ES102** | 0.847 |
| *cyclohexane* | **C_6_H_12_** | **HC38** | 0.958 | *(2E)-hexenyl acetate* | **C_8_H_14_O_2_** | **ES121** | 0.823 |
| *β-pinene* | **C_10_H_16_** | **TE112** | -0.460 | *propyl alcohol* | **C_3_H_8_O** | **AL18** | -0.256 |
| *3-octanone* | **C_8_H_16_O** | **KE96** | -0.493 | *3-methylbutanal* | **C_5_H_10_O** | **AD42** | -0.336 |

**Table S5. Results of agglomerative hierarchical clustering performed on PTR-TOF and GC-MS data. (***A***)** Variance decomposition (within/ between classes), class results (*B*) and distances between the class centroids (*C*) obtained from PTR-TOF data**. (***D***)** Variance decomposition (within/ between classes), class results (*E*) and distances between the class centroids (*F*) obtained from GC-MS data**.**

| **PTR-MS** | | **A** |  |  |  |  | **B** |  |  |  |  | **C** |  |  |  |  |  |  |  |  |
| --- | --- | --- | --- | --- | --- | --- | --- | --- | --- | --- | --- | --- | --- | --- | --- | --- | --- | --- | --- | --- |
| **Variance** | **Absolute** | **Percent** |  | **Classes** | **C1** | **C2** | **C3** |  |  | **C1** | **C2** | **C3** |  |  |  |  |  |  |  |  |
| *Within-class* | 11.15 | 0.48 |  | *Objects* | 8.00 | 6.00 | 3.00 |  | **C1** | 0.00 | 7.13 | 5.60 |  |  |  |  |  |  |  |  |
| *Between-classes* | 12.30 | 0.52 |  | *Within-class variance* | 13.12 | 7.54 | 13.25 |  | **C2** | 7.13 | 0.00 | 5.43 |  |  |  |  |  |  |  |  |
| ***Total*** | 23.44 | 1.00 |  |  | **ALqp** | **MScb** | **CApa** |  | **C3** | 5.60 | 5.43 | 0.00 |  |  |  |  |  |  |  |  |
|  |  |  |  |  | **ALpa** | **MSqb** | **CAsa** |  |  |  |  |  |  |  |  |  |  |  |  |  |
|  |  |  |  |  | **MSqp** | **MMsa** | **HRwd** |  |  |  |  |  |  |  |  |  |  |  |  |  |
|  |  |  |  | ***Samples*** | **MMcb** | **MMqc** |  |  |  |  |  |  |  |  |  |  |  |  |  |  |
|  |  |  |  |  | **CAqp** | **MMpa** |  |  |  |  |  |  |  |  |  |  |  |  |  |  |
|  |  |  |  |  | **SMwd** | **ISpa** |  |  |  |  |  |  |  |  |  |  |  |  |  |  |
|  |  |  |  |  | **SGwd** |  |  |  |  |  |  |  |  |  |  |  |  |  |  |  |
|  |  |  |  |  | **AQwd** |  |  |  |  |  |  |  |  |  |  |  |  |  |  |  |
|  |  |  |  |  |  |  |  |  |  |  |  |  |  |  |  |  |  |  |  |  |
| **GC-MS** | | **D** |  |  |  |  |  |  |  |  | **E** |  |  |  |  |  |  |  |  | **F** |
| **Variance** | **Absolute** | **Percent** |  | **Classes** | **C1** | **C2** | **C3** | **C4** | **C5** | **C6** | **C7** |  |  | **C1** | **C2** | **C3** | **C4** | **C5** | **C6** | **C7** |
| *Within-class* | 141.90 | 0.57 |  | *Objects* | 3.00 | 3.00 | 4.00 | 1.00 | 1.00 | 1.00 | 1.00 |  | **C1** | 0.00 | 14.53 | 12.27 | 23.21 | 22.96 | 27.28 | 21.36 |
| *Between-classes* | 105.85 | 0.43 |  | *Within-class variance* | 137.40 | 125.72 | 155.69 | 0.00 | 0.00 | 0.00 | 0.00 |  | **C2** | 14.53 | 0.00 | 12.68 | 23.31 | 19.86 | 27.86 | 20.59 |
| ***Total*** | 247.75 | 1.00 |  |  | **ALqp** | **MScb** | **MMcb** | **CApa** | **CAsa** | **SMwd** | **HRwd** |  | **C3** | 12.27 | 12.68 | 0.00 | 22.18 | 19.62 | 26.01 | 19.06 |
|  |  |  |  | ***Samples*** | **ALpa** | **MSqb** | **MMsa** |  |  |  |  |  | **C4** | 23.21 | 23.31 | 22.18 | 0.00 | 25.29 | 31.05 | 25.53 |
|  |  |  |  |  | **SGwd** | **MMqc** | **CAqp** |  |  |  |  |  | **C5** | 22.96 | 19.86 | 19.62 | 25.29 | 0.00 | 31.10 | 27.06 |
|  |  |  |  |  |  |  | **ISpa** |  |  |  |  |  | **C6** | 27.28 | 27.86 | 26.01 | 31.05 | 31.10 | 0.00 | 26.71 |
|  |  |  |  |  |  |  |  |  |  |  |  |  | **C7** | 21.36 | 20.59 | 19.06 | 25.53 | 27.06 | 26.71 | 0.00 |

**Table S6. Climatic data on the fruiting bodies sampling area for the five-year period 2012-2016**. Data were downloaded from climate stations close to sampling sites. **AL** (Alba Tanaro; https://www.arpa.piemonte.gov.it); **CA** (Stia monte; http://www.sir.toscana.it: TOS11000105); **IS** (Isernia Colle Vecchio; https://www.meteoisernia.net); **MS** (Sant’Angelo in Vado; http://84.38.48.145/sol/indexjs.php?lang=it); **SM** (San Miniato; http://www.sir.toscana.it: TOS01001491); **HR** (Pula; https://www.wunderground.com); **AQ** (L’Aquila; http://meteorema.aquila.infn.it); **SG** (San Gimignano; http://www.sir.toscana.it: TOS01001419). * = The data concerning the Marche region (two collecting areas: Mercatello sul Metauro and Sant'Angelo in Vado) have been reported only for one area because they are only 8 km away.

|  |  | **AL** | **CA** | **IS** | **MS, MM*** | **SM** | **HR** | **AQ** | **SG** |
| --- | --- | --- | --- | --- | --- | --- | --- | --- | --- |
| ***Altitude (m)*** |  | 172 | 838 | 600 | 360 | 102 | 84 | 680 | 306 |
| ***Temperature (°C)*** | ***Jan*** | 2.88 ± 0.92 | 4.42 ± 0.62 | 6.05 ± 1.08 | 4.93 ± 1.43 | 7.78 ± 0.98 | 6.03 ± 1.18 | 6.38 ± 2.92 | 8.50 ± 1.50 |
|  | ***Feb*** | 3.30 ± 2.49 | 3.54 ± 2.77 | 5.60 ± 1.97 | 4.59 ± 3.77 | 7.98 ± 2.58 | 5.99 ± 2.80 | 6.94 ± 4.13 | 7.64 ± 2.01 |
|  | ***Mar*** | 8.96 ± 1.85 | 7.44 ± 1.76 | 8.05 ± 0.37 | 8.05 ± 0.82 | 11.6 ± 1.39 | 9.25 ± 1.36 | 10.6 ± 4.47 | 11.52 ± 2.82 |
|  | ***Apr*** | 13.28 ± 0.78 | 11.04 ± 0.99 | 12.23 ± 1.09 | 11.86 ± 0.70 | 14.86 ± 0.60 | 12.41 ± 0.41 | 14.5 ± 3.91 | 14.78 ± 1.26 |
|  | ***May*** | 16.96 ± 1.14 | 13.4 ± 1.06 | 14.85 ± 1.00 | 14.82 ± 0.97 | 17.48 ± 0.77 | 15.93 ± 0.66 | 17.18 ± 3.28 | 17.24 ± 1.59 |
|  | ***Jun*** | 22.1 ± 0.84 | 18.26 ± 1.09 | 18.63 ± 0.53 | 19.42 ± 1.22 | 22.36 ± 1.06 | 21.11 ± 1.06 | 21.98 ± 5.02 | 22.28 ± 2.27 |
|  | ***Jul*** | 24.76 ± 1.89 | 21.62 ± 1.98 | 21.93 ± 1.94 | 22.46 ± 1.83 | 25.42 ± 1.69 | 24.42 ± 1.53 | 25.16 ± 5.21 | 25.34 ± 2.41 |
|  | ***Aug*** | 23.54 ± 1.20 | 21.34 ± 1.65 | 21.80 ± 0.88 | 21.45 ± 1.24 | 25.26 ± 1.65 | 23.75 ± 1.49 | 25.1 ± 5.18 | 25.24 ± 2.83 |
|  | ***Sep*** | 19.07 ± 0.94 | 17.00 ± 0.61 | 17.90 ± 0.53 | 17.18 ± 0.43 | 21.26 ± 0.62 | 19.41 ± 0.86 | 20.12 ± 3.78 | 20.64 ± 0.79 |
|  | ***Oct*** | 13.62 ± 1.14 | 13.10 ± 0.93 | 14.20 ± 0.93 | 13.51 ± 1.26 | 17.16 ± 0.99 | 14.83 ± 0.98 | 15.94 ± 3.28 | 16.52 ± 1.12 |
|  | ***Nov*** | 8.31 ± 0.95 | 8.88 ± 1.38 | 10.25 ± 0.85 | 9.61 ± 0.95 | 12.68 ± 0.93 | 11.14 ± 1.24 | 10.66 ± 2.89 | 12.00 ± 1.03 |
|  | ***Dec*** | 3.16 ± 1.27 | 5.86 ± 1.32 | 6.45 ± 0.50 | 4.62 ± 0.96 | 8.44 ± 1.06 | 6.81 ± 0.95 | 5.92 ± 3.03 | 8.82 ± 1.11 |
| ***Rainfall (mm)*** | ***Jan*** | 38.4 ± 15.67 | 131.64 ± 74.53 | 103.83 ± 45.51 | 93.36 ± 35.44 | 92.52 ± 81.46 | 65.79 ± 28.02 | 47.38 ± 24.1 | 79.20 ± 47.82 |
|  | ***Feb*** | 63.00 ± 55.46 | 129.60 ± 99.89 | 108.80 ± 21.15 | 150.48 ± 55.31 | 106.36 ± 84.24 | 75.31 ± 56.19 | 59.06 ± 17.94 | 100.12 ± 76.86 |
|  | ***Mar*** | 99.72 ± 48.97 | 138.68 ± 92.42 | 88.00 ± 47.63 | 113.28 ± 55.05 | 90.08 ± 72.59 | 54.80 ± 25.59 | 55.08 ± 45.58 | 68.68 ± 48.19 |
|  | ***Apr*** | 71.36 ± 47.79 | 89.12 ± 56.63 | 53.15 ± 28.81 | 91.36 ± 26.47 | 84.68 ± 37.75 | 32.00 ± 21.36 | 44.20 ± 20.25 | 63.88 ± 21.92 |
|  | ***May*** | 96.20 ± 42.16 | 139.72 ± 42.04 | 61.23 ± 32.01 | 131.6 ± 8.38 | 76.76 ± 42.20 | 24.23 ± 14.21 | 57.06 ± 31.25 | 60.88 ± 36.87 |
|  | ***Jun*** | 38.92 ± 36.23 | 57.80 ± 30.48 | 45.35 ± 39.79 | 72.36 ± 17.4 | 43.60 ± 25.53 | 35.97 ± 21.53 | 51.46 ± 41.11 | 39.88 ± 24.63 |
|  | ***Jul*** | 47.56 ± 35.56 | 57.40 ± 44.5 | 42.75 ± 29.9 | 50.84 ± 46.57 | 32.24 ± 59.13 | 26.47 ± 49.99 | 48.00 ± 26.37 | 35.44 ± 35.42 |
|  | ***Aug*** | 21.32 ± 20.38 | 38.88 ± 20.29 | 22.70 ± 24.28 | 69.12 ± 33.43 | 41.52 ± 32.53 | 42.72 ± 12.76 | 19.50 ± 13.84 | 31.16 ± 22.74 |
|  | ***Sep*** | 26.68 ± 22.58 | 68.56 ± 34.85 | 52.13 ± 44.41 | 89.16 ± 21.32 | 74.92 ± 36.38 | 61.67 ± 45.67 | 56.88 ± 23.88 | 63.48 ± 10.57 |
|  | ***Oct*** | 46.68 ± 29.23 | 156.56 ± 72.74 | 59.03 ± 27.66 | 123.40 ± 26.34 | 123.28 ± 50.88 | 67.30 ± 33.43 | 68.14 ± 39.85 | 134.72 ± 74.01 |
|  | ***Nov*** | 117.56 ± 86.24 | 218.84 ± 106.83 | 106.00 ± 11.44 | 196.36 ± 95.95 | 103.92 ± 59.7 | 80.31 ± 39.63 | 74.40 ± 34.95 | 126.96 ± 68.77 |
|  | ***Dec*** | 54.45 ± 36.60 | 56.24 ± 56.64 | 46.75 ± 77.37 | 47.60 ± 50.53 | 51.80 ± 52.59 | 17.88 ± 18.17 | 24.68 ± 23.8 | 42.80 ± 51.42 |

**Table S7. List of identified compounds through MS analysis**. AL= Alcohols; AD= Aldehydes; AR= Aromatic compounds; ES= Esters; HC= Hydrocarbons; KE= Ketones; OT= Others; SU=Sulfur containing compounds; TE= Terpenes. The average masses data were downloaded from chemspider website (www.chemspider.com).

| **Unified code** | *Average mass* | **Compounds** | Chemical class | PTR-MS data | GC-MS data | **Unified code** | *Average mass* | **Compounds** | Chemical class | PTR-MS data | GC-MS data |
| --- | --- | --- | --- | --- | --- | --- | --- | --- | --- | --- | --- |
| **1** | **26.037** | acetylene | **HC** | **x** |  | **111** | **136.234** | α-pinene | **TE** |  | **x** |
| **2** | **30.026** | formaldehyde | **AD** | **x** |  | **112** | **136.234** | β-pinene | **TE** |  | **x** |
| **3** | **32.042** | methanol | **AL** | **x** |  | **113** | **136.234** | 2,2-dimethyl-5-methylene norbornane | **TE** |  | **x** |
| **4** | **40.064** | alkyl fragment | **HC** | **x** |  | **114** | **136.238** | α-fenchene | **TE** |  | **x** |
| **5** | **42.037** | alkyl fragment (ethenone) | **KE** | **x** |  | **115** | **136.240** | myrcene | **TE** |  | **x** |
| **6** | **43.088** | alkyl fragment (propene) | **HC** | **x** |  | **116** | **136.240** | α-terpinene | **TE** |  | **x** |
| **7** | **44.053** | acetic aldehyde | **AD** | **x** | **x** | **117** | **138.207** | 2-pentyl-furan | **OT** |  | **x** |
| **8** | **46.092** | methanethial | **SU** | **x** |  | **118** | **138.207** | ligustral | **AD** |  | **x** |
| **9** | **46.068** | ethanol | **AL** | **x** |  | **119** | **138.207** | trivertal | **AD** |  | **x** |
| **10** | **48.107** | methanethiol | **SU** | **x** |  | **120** | **140.266** | 1-decene | **HC** |  | **x** |
| **11** | **52.075** | C4 compound | **HC** | **x** |  | **121** | **142.196** | (2E)-hexenyl acetate | **ES** |  | **x** |
| **12** | **54.090** | alkyl fragment | **HC** | **x** |  | **122** | **142.239** | *n*-nonanal | **AD** |  | **x** |
| **13** | **56.063** | 2-propenal | **AD** | **x** |  | **123** | **142.239** | diisobutyl ketone | **KE** |  | **x** |
| **14** | **56.106** | alkyl fragment (1-butene) | **HC** | **x** |  | **124** | **142.239** | 4-nonanone | **KE** |  | **x** |
| **15** | **58.102** | Sulphur compounds (acetylenethiol / thiirene) | **SU** | **x** |  | **125** | **142.239** | 2-nonanone | **KE** |  | **x** |
| **16** | **58.079** | acetone | **KE** | **x** | **x** | **126** | **142.280** | *n*-decane | **HC** |  | **x** |
| **17** | **60.052** | acetic acid | **OT** | **x** |  | **127** | **144.211** | isobutyl-isobutyrate | **ES** |  | **x** |
| **18** | **60.100** | propyl alcohol | **AL** |  | **x** | **128** | **144.211** | butyroin | **KE** |  | **x** |
| **19** | **62.134** | dimethyl sulfide | **SU** | **x** | **x** | **129** | **144.255** | nonylol | **AL** |  | **x** |
| **20** | **64.041** | methanetriol | **OT** | **x** |  | **130** | **144.255** | 2-nonanol | **AL** |  | **x** |
| **21** | **66.101** | 3 penten-1-yne | **HC** | **x** |  | **131** | **152.233** | camphor | **TE** |  | **x** |
| **22** | **67.089** | pyrrole | **OT** | **x** |  | **132** | **154.249** | eucalyptol | **TE** |  | **x** |
| **23** | **68.074** | furan | **AR** | **x** |  | **133** | **154.249** | (4Z)-decenal | **AD** |  | **x** |
| **24** | **68.117** | 2-methyl-1,3- butadiene | **TE** | **x** |  | **134** | **154.249** | linalool | **TE** |  | **x** |
| **25** | **70.090** | (2E)-butenal | **AD** | **x** | **x** | **135** | **156.222** | γ-nonalactone | **KE** |  | **x** |
| **26** | **70.133** | alkyl fragment | **HC** | **x** |  | **136** | **156.265** | α-dihydro-terpineol | **TE** |  | **x** |
| **27** | **72.063** | acrylic acid | **OT** | **x** |  | **137** | **156.265** | (4Z)-decen-1-ol | **AL** |  | **x** |
| **28** | **72.106** | butanone | **KE** | **x** | **x** | **138** | **156.265** | 2-decanone | **KE** |  | **x** |
| **29** | **74.079** | methyl acetate | **ES** | **x** |  | **139** | **156.308** | *n*-undecane | **HC** |  | **x** |
| **30** | **74.122** | isobutyl alcohol | **AL** |  | **x** | **140** | **158.195** | *n*-butyric anhydride | **OT** |  | **x** |
| **31** | **76.161** | 1-propanethiol | **SU** | **x** |  | **141** | **158.195** | tetrahydrofurfuryl propionate | **ES** |  | **x** |
| **32** | **78.157** | Sulphur compounds (dithioformic acid) | **SU** | **x** |  | **142** | **158.238** | Isobutyl pentanoate | **ES** |  | **x** |
| **33** | **78.133** | dimethyl sulfoxide | **SU** | **x** |  | **143** | **168.320** | 1-dodecene | **HC** |  | **x** |
| **34** | **80.128** | alkyl fragment (hexenals/hexenols/terpenoids) | **HC** | **x** |  | **144** | **170.292** | 2-undecanone | **KE** |  | **x** |
| **35** | **82.101** | 2-methylfuran | **AR** | **x** |  | **145** | **170.292** | *n*-undecanal | **AD** |  | **x** |
| **36** | **82.144** | C6 compounds | **HC** | **x** |  | **146** | **172.265** | 3-acetoxyoctane | **ES** |  | **x** |
| **37** | **84.116** | 3-penten-2-one | **KE** | **x** |  | **147** | **172.265** | hexyl isobutyrate | **ES** |  | **x** |
| **38** | **84.160** | cyclohexane | **HC** |  | **x** | **148** | **172.265** | 3-methyl pentyl-Isobutyrate | **ES** |  | **x** |
| **39** | **86.089** | vinyl acetate | **ES** | **x** | ***x*** | **149** | **176.296** | diethyl acetal | **OT** |  | **x** |
| **40** | **86.132** | 3-pentanone | **KE** | **x** | ***x*** | **150** | **182.346** | 1-tridecene | **HC** |  | **x** |
| **41** | **86.130** | 2-pentanone | **KE** |  | **x** | **151** | **184.275** | allyl caprylate | **ES** |  | **x** |
| **42** | **86.130** | 3-methylbutanal | **AD** |  | **x** | **152** | **184.275** | (Z)-3-nonenyl acetate | **ES** |  | **x** |
| **43** | **86.132** | 2-methylbutanal | **AD** |  | **x** | **153** | **184.275** | (E)-2-nonen-1-yl acetate | **ES** |  | **x** |
| **44** | **88.171** | allyl methyl sulfide | **SU** | **x** |  | **154** | **184.275** | (6Z)-6-nonenyl acetate | **ES** |  | **x** |
| **45** | **88.105** | 3-hydroxy-2-butanone | **KE** | **x** |  | **155** | **186.251** | allyl 3-methyl butoxyacetate | **ES** |  | **x** |
| **46** | **88.148** | isopentyl alcohol | **AL** |  | **x** | **156** | **186.291** | 3,5,5-trimethylhexyl-acetate | **ES** |  | **x** |
| **47** | **88.148** | sec-pentanol | **AL** |  | **x** | **157** | **186.291** | heptyl-Isobutyrate | **ES** |  | **x** |
| **48** | **88.148** | tert-butyl methyl ether | **OT** |  | **x** | **158** | **186.334** | dodecanol | **AL** |  | **x** |
| **49** | **88.148** | *sec*-butyl-carbinol | **AL** |  | **x** | **159** | **198.302** | allyl 3,5,5-trimethyl hexanoate | **ES** |  | **x** |
| **50** | **90.121** | 2-3-butanediol | **AL** | **x** |  | **160** | **198.345** | 2-tridecanone | **KE** |  | **x** |
| **51** | **92.183** | 1,2-dithietane | **SU** | **x** |  | **161** | **212.330** | (E)-2-methyl-2-butenoic acid 1-ethylhexyl ester | **ES** |  | **x** |
| **52** | **92.160** | 2-methylmercaptoethanol | **SU** | **x** |  | **162** | **214.344** | methyl-dodecanoate | **ES** |  | **x** |
| **53** | **92.138** | toluene | **AR** | **x** | **x** | **163** | **220.307** | α-,α-dimethyl-phenethyl butyrate | **ES** |  | **x** |
| **54** | **94.133** | dimethyl sulfone | **SU** | **x** |  |  |  |  |  |  |  |
| **55** | **94.199** | dimethyl disulfide | **SU** | **x** | **x** |  |  |  |  |  |  |
| **56** | **96.084** | furfural (2-furancarboxaldehyde) | **AD** | **x** |  |  |  |  |  |  |  |
| **57** | **96.127** | 2,5-dimethyl-furan | **OT** | **x** | **x** |  |  |  |  |  |  |
| **58** | **98.100** | 4-methyl-(5H)-furan-2-one | **KE** | **x** |  |  |  |  |  |  |  |
| **59** | **98.143** | 4-methyl-3-pentene-2-one | **KE** | **x** | **x** |  |  |  |  |  |  |
| **60** | **98.143** | 4-methylpent-2-enal | **AD** |  | **x** |  |  |  |  |  |  |
| **61** | **100.116** | acetylpropionyl | **KE** | **x** | **x** |  |  |  |  |  |  |
| **62** | **100.159** | *n*-hexanal | **AD** |  | **x** |  |  |  |  |  |  |
| **63** | **100.159** | 2-methyl-pentanal | **AD** |  | **x** |  |  |  |  |  |  |
| **64** | **100.182** | 2-methyl-4,5-dihydrothiophene | **SU** | **x** |  |  |  |  |  |  |  |
| **65** | **102.133** | ethynylbenzene | **AR** | **x** |  |  |  |  |  |  |  |
| **66** | **102.132** | 4-hydroxy-3-methyl-2-butanone | **KE** | **x** |  |  |  |  |  |  |  |
| **67** | **102.132** | methyl isobutyrate | **ES** |  | **x** |  |  |  |  |  |  |
| **68** | **102.175** | *n*-hexanol | **AL** |  | **x** |  |  |  |  |  |  |
| **69** | **104.171** | 3-methylthio-propionaldehyde | **SU** | **x** | **x** |  |  |  |  |  |  |
| **70** | **104.104** | 4-hydroxybutanoic acid | **OT** | **x** |  |  |  |  |  |  |  |
| **71** | **104.150** | styrene | **AR** |  | **x** |  |  |  |  |  |  |
| **72** | **106.144** | 2-methylthioacetic acid | **SU** | **x** |  |  |  |  |  |  |  |
| **73** | **106.165** | ethylbenzene | **AR** | **x** | ***x*** |  |  |  |  |  |  |
| **74** | **106.122** | benzaldehyde | **AR** |  | **x** |  |  |  |  |  |  |
| **75** | **108.161** | 2-ethynylthiophene | **SU** | **x** |  |  |  |  |  |  |  |
| **76** | **108.138** | anisole | **AR** | **x** |  |  |  |  |  |  |  |
| **77** | **108.226** | bis(methylthio)methane | **SU** |  | **x** |  |  |  |  |  |  |
| **78** | **110.111** | 2-acetylfuran | **OT** | **x** | **x** |  |  |  |  |  |  |
| **79** | **112.170** | (2E)-heptenal | **AD** |  | **x** |  |  |  |  |  |  |
| **80** | **114.166** | 2-methyl-3-furanthiol | **SU** | **x** |  |  |  |  |  |  |  |
| **81** | **114.142** | 3,5-dimethyldiidro-2(3H)-furanone | **KE** | **x** |  |  |  |  |  |  |  |
| **82** | **114.186** | 2-heptanone | **KE** |  | **x** |  |  |  |  |  |  |
| **83** | **114.186** | *n*-heptanal | **AD** |  | **x** |  |  |  |  |  |  |
| **84** | **116.201** | *n*-heptanol | **AL** | **x** | **x** |  |  |  |  |  |  |
| **85** | **118.130** | ethyl-lactate | **ES** |  | **x** |  |  |  |  |  |  |
| **86** | **120.149** | phenylacetaldehyde | **AR** |  | **x** |  |  |  |  |  |  |
| **87** | **122.160** | phenethyl alcohol | **AR** |  | **x** |  |  |  |  |  |  |
| **88** | **124.137** | 2-acetyl-5-methylfuran | **KE** | **x** | **x** |  |  |  |  |  |  |
| **89** | **126.110** | methyl 2-furancarboxylate | **ES** | **x** |  |  |  |  |  |  |  |
| **90** | **126.196** | 3-octen-2-one | **KE** | **x** | **x** |  |  |  |  |  |  |
| **91** | **126.153** | 2-acetyl-cyclopentanone | **KE** |  | **x** |  |  |  |  |  |  |
| **92** | **126.196** | 6-methyl-hept-5-en-2-one | **KE** |  | **x** |  |  |  |  |  |  |
| **93** | **126.196** | (2E)-octenal | **AD** |  | **x** |  |  |  |  |  |  |
| **94** | **126.260** | dimethyl trisulfide | **SU** |  | **x** |  |  |  |  |  |  |
| **95** | **128.169** | acetyl isovaleryl | **KE** |  | **x** |  |  |  |  |  |  |
| **96** | **128.212** | 3-octanone | **KE** |  | **x** |  |  |  |  |  |  |
| **97** | **128.212** | 2-octanone | **KE** |  | **x** |  |  |  |  |  |  |
| **98** | **128.212** | 3Z-octenol | **AL** |  | **x** |  |  |  |  |  |  |
| **99** | **128.212** | *n*-octanal | **AD** |  | **x** |  |  |  |  |  |  |
| **100** | **128.220** | 1-octen-3-ol | **AL** |  | **x** |  |  |  |  |  |  |
| **101** | **128.255** | *n*-nonane | **HC** |  | **x** |  |  |  |  |  |  |
| **102** | **130.140** | ethyl-acetoacetate | **ES** |  | **x** |  |  |  |  |  |  |
| **103** | **130.210** | S-allyl-thiopropionate | **SU** |  | **x** |  |  |  |  |  |  |
| **104** | **130.230** | ethyl-hexanol | **AL** |  | **x** |  |  |  |  |  |  |
| **105** | **130.230** | *n*-octanol | **AL** |  | **x** |  |  |  |  |  |  |
| **106** | **134.218** | *para*-cymene | **TE** |  | **x** |  |  |  |  |  |  |
| **107** | **136.234** | γ-terpinene | **TE** |  | **x** |  |  |  |  |  |  |
| **108** | **136.234** | sabinene | **TE** |  | **x** |  |  |  |  |  |  |
| **109** | **136.234** | limonene | **TE** |  | **x** |  |  |  |  |  |  |
| **110** | **136.234** | terpinolene | **TE** |  | **x** |  |  |  |  |  |  |

**Table S8. (A) Compound classes and their relative contribution to PCA dimensions related to the analysis of antioxidant power**. **(B) Compounds classes and their relative contribution to PCA dimensions related to electronic nose analysis**. Each dimension of a multivariate analysis can be described by the variables that are used to construct the factorial axes. ∆Q indicates the sensor used for the measurements. ^l^ Compound and variable classes are sorted according to their relative contribution to dimension 1.

A

| **Compound class^l^** | **Dimension 1** | **Dimension 2** |
| --- | --- | --- |
| *Total antioxidant power* | **36.12** | **1.79** |
| *Hydrophilic antioxidant power* | **35.70** | **0.52** |
| *Total phenols* | **18.56** | **21.29** |
| *Lipophilic antioxidant power* | **8.58** | **33.61** |
| *Total glutathione* | **1.03** | **2.48** |
| *Total Ascorbate* | **0.01** | **40.31** |

B

| **Variable class^l^** | **Dimension 1** | **Dimension 2** |
| --- | --- | --- |
| *∆Q5* | **22.54** | **3.04** |
| *∆Q7* | **22.45** | **0.95** |
| *∆Q8* | **22.08** | **4.46** |
| *∆Q3* | **19.69** | **5.97** |
| *∆Q4* | **13.24** | **85.58** |

References

55 Mancuso, S. *et al.* Soil volatile analysis by proton transfer reaction-time of flight mass spectrometry (PTR-TOF-MS). *Applied Soil Ecology* **86**, 182-191 (2015).

56 Taiti, C. *et al.* Nashi or Williams pear fruits? Use of volatile organic compounds, physicochemical parameters, and sensory evaluation to understand the consumer’s preference. *European Food Research and Technology* **243**, 1917-1931 (2017).

57 Lindinger, W., Hansel, A. & Jordan, A. On-line monitoring of volatile organic compounds at pptv levels by means of proton-transfer-reaction mass spectrometry (PTR-MS) medical applications, food control and environmental research. *International Journal of Mass Spectrometry and Ion Processes* **173**, 191-241 (1998).

58 Herbig, J. *et al.* On-line breath analysis with PTR-TOF. *Journal of breath research* **3**, 027004 (2009).

59 Fabris, A. *et al.* PTR‐TOF‐MS and data‐mining methods for rapid characterisation of agro‐industrial samples: influence of milk storage conditions on the volatile compounds profile of Trentingrana cheese. *Journal of mass spectrometry* **45**, 1065-1074 (2010).

60 Cappellin, L. *et al.* PTR-ToF-MS and data mining methods: a new tool for fruit metabolomics. *Metabolomics* **8**, 761-770 (2012).

61 Re, R. *et al.* Antioxidant activity applying an improved ABTS radical cation decolorization assay. *Free radical biology and medicine* **26**, 1231-1237 (1999).

62 Waterhouse, A. L. Determination of total phenolics. *Current protocols in food analytical chemistry* (2002).

63 Foyer, C. H., Pellny, T. K., Locato, V. & De Gara, L. Analysis of redox relationships in the plant cell cycle: determinations of ascorbate, glutathione and poly (ADPribose) polymerase (PARP) in plant cell cultures. *Redox-Mediated Signal Transduction: Methods and Protocols*, 193-209 (2009).

64 De Pinto, M., Francis, D. & De Gara, L. The redox state of the ascorbate-dehydroascorbate pair as a specific sensor of cell division in tobacco BY-2 cells. *Protoplasma* **209**, 90-97 (1999).

65 Santonico, M., Pennazza, G., Grasso, S., D'Amico, A. & Bizzarri, M. Design and test of a biosensor-based multisensorial system: A proof of concept study. *Sensors* **13**, 16625-16640 (2013).

66 Pantalei, S. *et al.* Enhanced sensory properties of a multichannel quartz crystal microbalance coated with polymeric nanobeads. *Sensors* **7**, 2920-2928 (2007).

67 Escofier, B. & Pages, J. Multiple factor analysis (AFMULT package). *Computational statistics & data analysis* **18**, 121-140 (1994).

68 Ward Jr, J. H. Hierarchical grouping to optimize an objective function. *Journal of the American statistical association* **58**, 236-244 (1963).

69 Podani, J. *Introduction to the exploration of multivariate biological data*. (Backhuys Publishers, 2000).
